# Therapeutic potential of the human endogenous retroviral envelope protein HEMO: a pan‐cancer analysis

**DOI:** 10.1002/1878-0261.13069

**Published:** 2021-10-11

**Authors:** Amélie Kasperek, Anthony Béguin, Olivia Bawa, Kévin De Azevedo, Bastien Job, Christophe Massard, Jean‐Yves Scoazec, Thierry Heidmann, Odile Heidmann

**Affiliations:** ^1^ CNRS UMR 9196 Laboratory of Molecular Physiology and Pathology of Endogenous and Infectious Retroviruses Gustave Roussy University Paris‐Saclay Villejuif France; ^2^ PETRA Platform, AMMICa CNRS‐UMS 3655 and INSERM‐US23 Gustave Roussy University Paris‐Saclay Villejuif France; ^3^ Bioinformatic Core Facility AMMICa CNRS‐UMS 3655 and INSERM‐US23 Gustave Roussy University Paris‐Saclay Villejuif France; ^4^ Drug Development Department (DITEP) Gustave Roussy University Paris‐Saclay Villejuif France; ^5^ University Paris‐Saclay, Faculty of Medicine Le Kremlin Bicêtre France; ^6^ Department of Pathology Gustave Roussy Villejuif France

**Keywords:** cancer, ERVMER34‐1, HEMO, HERV, TCGA, Wnt/β‐catenin

## Abstract

Human endogenous retroviruses represent approximately 8% of our genome. Most of these sequences are defective except for a few genes such as the ancestral retroviral *HEMO* envelope gene (Human Endogenous MER34 ORF), recently characterized by our group. In this study, we characterized transcriptional activation of *HEMO* in primary tumors from The Cancer Genome Atlas (TCGA) and in metastatic tumors from a Gustave Roussy cohort. Pan‐cancer detection of the HEMO protein in a series of patient samples validated these results. Differential gene expression analysis in various TCGA datasets revealed a link between *HEMO* expression and activation of Wnt/β‐catenin signaling, in particular in endometrial cancer. Studies on cell models led us to propose that the Wnt/β‐catenin pathway could act as an upstream regulator of this retroviral endogenous sequence in tumor condition. Characterization of transcriptomic profiles of both HEMO^Low^ and HEMO^High^ tumors suggested that activation of *HEMO* is negatively associated with immune response signatures. Taken together, these results highlight that HEMO, as an endogenous retroviral envelope protein specifically expressed in tumors, represents a promising tumor biomarker and therapeutic target.

## Graphical abstract

Human endogenous retroviruses represent approximately 8% of our genome. Among these sequences, HEMO (Human Endogenous MER34 ORF) is the oldest retroviral envelope gene, endogenized 100 million years ago. Using *in silico* and experimental pan‐cancer approaches, we showed that the HEMO protein is a tumor biomarker whose expression can be regulated by the Wnt/β‐catenin pathway.

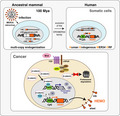

**Abbreviations**
ADC: adenocarcinoma
APC: adenomatosis polyposis coli
C: non-tumor control adjacent tissues
CTNNB1: catenin beta 1
ERVMER34‐1: endogenous retrovirus medium‐reiteration‐family‐34 member 1
FFPE: formalin‐fixed, paraffin‐embedded
GO: gene ontology
GSEA: gene set enrichment analysis
GTEx: Genotype‐Tissue Expression
HEMO: human endogenous MER34 ORF
Her2: human epidermal growth factor receptor 2
HERV: human endogenous retroviruses
Kid-N: kidney normal tissues
MOSCATO: molecular screening for cancer treatment optimization
N: normal tissues
SCC: squamous cell carcinoma
T: tumor tissues
TCGA: The Cancer Genome Atlas

## 1 Introduction

Human endogenous retroviruses (HERVs) originate from ancient retroviral germline invasions by infectious retroviruses, that recurrently occurred in vertebrates, since their origin more than 450 million years ago [1, 2, 3, 4, 5]. Infection and entry of the retrovirus into the cell are usually driven by an interaction between the retroviral envelope protein and a membrane protein used as a receptor on the target cell. The RNA retroviral genome is then reverse‐transcribed and integrated as a DNA proviral copy into the chromosome, with the retroviral *gag*‐*pol*‐*env* genes flanked by two LTR sequences (5′ and 3′ long terminal repeats). These relatively frequent events in a retroviral infectious cycle usually concern somatic cells and facilitate spreading of the retroviruses to other cells and individuals, the most representative of current infectious retroviruses in humans being HIV and HTLV‐I retroviruses. However, infections can also target germline cells in adults or embryonic cells at an early stage *in utero* and consequently fix the retroviral genome in cells of the descendants, as an endogenous retrovirus (ERV). Although rare at the individual level, these events have accumulated during the course of vertebrate evolution. Endogenized retroviral sequences were thereafter transmitted in a Mendelian way. Today, in humans, these stably inherited sequences occupy up to 8% of the genome [6] and are classified by sequence homology into HERV families, each corresponding to the initial infectious retrovirus [7]. As a result of genetic alterations, the majority of HERVs are highly defective, frequently recombined as solo LTRs or even scattered as fragments of retroviral sequences in the chromosomes. Nevertheless, due to selective advantage of some retroviral functions for the host physiology, a few retroviral envelope (*env*) genes retained their coding capacity, being ‘coopted’ by the host and distinctively preserved from genetic drift. This can be illustrated by the well‐known example of syncytins, essential in mammalian placental development because they drive cellular fusion in syncytiotrophoblast formation and possibly influence feto‐maternal immune tolerance [8, 9, 10, 11].

Besides their expression during embryogenesis, *HERV* sequences (as coding elements or fragments of noncoding sequences) are on the whole silenced by epigenetic mechanisms in adult tissues [12, 13, 14]. Transcriptional activation of these sequences has been reported in several diseases such as cancer [15, 16, 17], autoimmune [18, 19, 20], or neurological disorders [21, 22, 23], underlying the ambivalent role of HERVs. Diverse external and host factors have been proposed as causative agents of this dysregulation [24]. Host factors include epigenetic modifications of DNA or histone, as well as cellular transcription factors since LTR sequences contain numerous transcription factor binding sites [25].

Contribution of several signaling pathways in tumorigenesis has been reported, including Wnt signaling, known for its critical role in cell–cell communication during embryonic development and adult tissue homeostasis [26]. Hyperactivation of the canonical Wnt/β‐catenin pathway has been associated with human diseases such as cancer. Indeed, alterations of its components leading to aberrant signaling are found in many solid and hematological tumors [27, 28, 29, 30].

Our group previously reported the characterization of the *HEMO* gene (*H*uman *E*ndogenous *M*Edium‐Reiteration‐frequency‐family‐34 *O*RF, or *H*uman *E*RV*M*ER34 *O*RF) which is part of a MER34 provirus, endogenized about 100 million years ago. At that time, infection of an ancestral mammal by a ‘MER34 retrovirus’ led to the integration of several proviral copies as a multigene family. All the MER34 proviral *gag*, *pol, and env* sequences became defective, except for one envelope gene that was kept under selection pressure until the present time, further suggesting a beneficial role during mammal evolution [31]. This integration event took place long before the integration events of the two human *syncytin‐1* and *syncytin‐2* genes, which were endogenized in the primate lineage less than 50 million years ago. Therefore, the *HEMO* gene is the oldest captured and still full‐length endogenous retroviral envelope (*env*) gene identified in the human genome (GRCh38, Ensembl annotation: ERVMER34‐1). Due to genomic alterations, *HEMO* lost its initial 5′ LTR promoter and is transcribed from a cellular CpG island located near the *env* gene. Remarkably, HEMO is expressed at high levels in placenta and stem cells and is a membrane protein that can be secreted into the blood as a shed protein [31].

In this study, we analyzed the expression of the endogenous retroviral sequence *HEMO* in primary and metastatic tumors by combining *in silico* analyses of RNAseq data and pan‐cancer detection at the protein level in patient samples. We also investigated its regulation and proposed the Wnt/β‐catenin pathway as an upstream regulator of this retroviral endogenous sequence in tumor condition.

## 2 Materials and methods

### 2.1 RNAseq datasets collection

Uniformly processed RNAseq data from GTEx (Genotype‐Tissue Expression project) for normal tissues (N: 5825 samples) and from TCGA (The Cancer Genome Atlas) for human tumor (T: 10181 samples) and non‐tumor control adjacent tissues (C: 722 samples) from a total of 33 different anatomic sites were downloaded from Recount2 resource (https://jhubiostatistics.shinyapps.io/recount/) [32].

Gene expression was quantified and reported in TPM unit (Transcripts Per Million of reads). Profile of *HEMO* expression was obtained using ENSEMBL_Id ENSG00000226887 (ERVMER34‐1). TCGA clinical data including tumor stage, grade, histological type, and molecular subtype were downloaded using r tcgabiolinks [33]. Corresponding mutation data were retrieved from cBioPortal for Cancer Genomics (http://www.cbioportal.org). Downstream transcriptomic data analyses were performed on tumor samples for which tumor nuclei exceeds 60%. RNAseq data (644 samples) collected from the clinical trial MOSCATO (MOlecular Screening for CAncer Treatment Optimization) led by Gustave Roussy were also used in our study [34]. Tumor cellularity of these samples ranged from 10% to 95%. Read quality control was performed with Trim galore (version 0.4.4) (https://www.bioinformatics.babraham.ac.uk/projects/trim_galore/), with the settings ‘‐q 20 ‐‐stringency 3 ‐‐gzip ‐‐length 20 ‐‐paired’. Afterward, the reads were pseudo‐mapped to the human transcriptome (GENCODE v27) with kallisto (version 0.44.0) [35] quant mode with the settings ‘‐‐bias ‐‐rf‐stranded’. Finally, to get gene‐level expression results, the package tximport (version 1.16.0) [36] was used.

### 2.2 Differential gene expression analysis

deseq2 R package (v1.26) was used to perform differential gene expression analysis between HEMO^High^ and HEMO^Low^ tumors. Samples of TCGA datasets were considered as HEMO^High^ or HEMO^Low^ if their expression level were, respectively, superior to 90th percentile or inferior to 10th percentile of global *HEMO* expression in the dataset. Eight cohorts with more than 300 samples were selected for differential gene expression analysis (BLCA, BRCA, CESC, COAD, HNSC, LUAD, LUSC, and UCEC). We corrected for the platform as a source of batch effect in differential gene expression analysis. Low‐expressed genes were filtered out before analysis. Differentially expressed genes were selected using the following cutoffs: FDR‐adjusted (Benjamini and Hochberg procedure) *P*‐value < 0.01, log2 fold change (|log2FC|) > 1.5, and ‘baseMean’ (the per‐gene mean value of normalized counts) > 50.

### 2.3 Gene set enrichment analysis

Gene set enrichment analysis was performed using r (v3.6.3) package clusterprofiler (v3.14.3). Genes ranking was performed using the decreasing log2FC value. This ranked list and gene sets from the REACTOME and GO (Gene Ontology) Biological Process databases were, respectively, retrieved from the C2 and C5 MsigDB v7.2 collections using the msigdbr (v7.2.1) package, and provided for the analysis carried out with 10^6^ permutations. A gene set was considered significantly enriched when adjusted *P*‐value was < 0.05.

### 2.4 Biological samples

All patient samples were obtained with written informed consent. The study methodologies conformed to the standards set by the Declaration of Helsinki. The study methodologies were approved by the local ethics committee. Formalin‐fixed, paraffin‐embedded (FFPE), and frozen samples of tumor and non‐tumor control adjacent tissues were obtained from the Biological Resource Centre (BB‐0033‐00074) and the Department of Pathology and Laboratory Medicine of GRCC (Gustave Roussy Cancer Campus/Research Agreements RT09916 and RT14017).

### 2.5 Immunohistochemistry assays

Sections (4 μm) of FFPE tumor, and non‐tumor adjacent tissues were deparaffinized in xylene and rehydrated. We used Bond Leica automated immunostainer (Leica Microsystèmes, Nanterre, France) for HEMO immunostaining and Ventana Benchmark Ultra (Roche Diagnostics, Meylan, France) automated immunostainer for cytokeratins 5/6, CDX2, or p63/HEMO immunostaining. Preparation and staining steps are summarized in Table S1. For all protocols, sections were counterstained with hematoxylin. A single representative whole tumor or normal tissue section from each patient was digitized using a slide scanner (VS120; Olympus Life Science, Waltham, MS, USA).

Detailed protocols of immunohistochemistry assays (Table S1) are provided in Supporting Information.

### 2.6 Cell lines, culture conditions, and FH535 treatment

Colorectal adenocarcinoma cell lines Caco‐2, HCT116, and SW480 were kindly gifted by F. Jaulin (Gustave Roussy). Lung cell lines NCIH520 (squamous carcinoma), HCC827 (adenocarcinoma), and ovary cell line OVMANA (clear cell adenocarcinoma) were kindly gifted by L. Friboulet (Gustave Roussy). Cells were grown at 37 °C with 5% CO_2_ in DMEM for Caco‐2 and SW480, Mc Coy's 5A medium for HCT116, and RPMI for NCIH520, HCC827, and OVMANA, supplemented with 10% heat‐inactivated fetal bovine serum, 100 μg·mL^−1^ streptomycin, and 100 U·mL^−1^ penicillin (all reagents were from Thermo Fisher Scientific, Illkirch, France). FH535 was purchased from Sigma‐Aldrich (St Quentin‐Fallavier, France) and dissolved in DMSO at 50 mm. Cells were seeded in 12‐well plates (1 × 10^5^ and 2 × 10^5^ cells/well for Caco‐2 and NCIH520) and treated 24 h after seeding with 40 μm of FH535 for 72 and 24 h for Caco‐2 and NCIH520, respectively.

### 2.7 siRNA transfection

Caco‐2 and NCIH520 cells were seeded as described above and transfected 24 h after seeding using Lipofectamine™ RNAiMAX reagent (Thermo Fisher Scientific) and CTNNB1 or Non‐targeting siRNA (SMART pool, ON‐TARGET Plus; Horizon Discovery, Cambridge, UK). Transfected cells were grown 2 days before analysis.

### 2.8 RNA extraction and RT‐qPCR

For RNA extraction from tissues, OCT‐frozen tumor and adjacent non‐tumor samples were sectioned with a cryostat. Non‐OCT‐frozen samples were disrupted with a mortar. 10 sections of 50 μm or 20–30 mg of tissue fragment were used. After mechanical disruption with glass beads, RNA extraction was performed with ReliaPrep™ RNA Tissue Miniprep System (Promega, Charbonnières‐les‐Bains, France). For cell lines, RNeasy Isolation Kit (Qiagen, Courtaboeuf, France) was used according to the manufacturer's instructions and treated with DNase I (Ambion, Thermo Fisher Scientific). RNA quality and concentration were assessed using a NanoDrop ND‐1000 spectrophotometer (Thermo Fisher Scientific). Reverse transcription was performed with 1 μg RNA using M‐MLV reverse transcriptase and random hexamers (Thermo Fisher Scientific). RT‐qPCR experiments were run on an ABI Prism 7000 sequence detection system with SYBR green PCR master mix reagent (Qiagen) and specific primers (Table S2). All analyses were carried out in triplicate, and transcript levels were normalized to the level of housekeeping gene RPLP0 using the ΔΔCT method.

### 2.9 Protein extraction and western blot analysis

For protein extraction from tissues, 50 mg of frozen tumor or adjacent non‐tumor samples was quickly lysed by mechanical disruption with glass beads in 200 μL RIPA buffer (Thermo Fisher Scientific) supplemented with Halt™ Protease Inhibitor Cocktail (Thermo Fisher Scientific). For protein extraction of cell lines, cells were briefly washed with PBS and then lysed in IP lysis buffer (25 mm Tris/HCl pH 7.4, 150 nm NaCl, 1% NP‐40, 1 mm EDTA) supplemented with Halt™ Protease Inhibitor Cocktail (Thermo Fisher Scientific). After removing cell debris by a centrifugation step (10 min, 4 °C at 14 000 ***g***), protein lysates were titrated with Pierce™ BCA Protein Assay Kit (Thermo Fisher Scientific). Samples or cell lysates were analyzed by SDS/PAGE on gradient precast gels under reducing conditions (NuPAGE™ Novex 4–12% Bis‐Tris gels; Thermo Fisher Scientific) and transferred onto nitrocellulose membranes using a dry transfer system (iBlot2; Thermo Fisher Scientific). After blocking in PBS containing 0.1% Tween‐20, and 5% non‐fat milk, membranes were incubated overnight at 4 °C with primary antibodies (anti‐HEMO mouse mAb 2F7 [31]; anti‐β‐catenin, BD biosciences, Le Pont de Claix, France, #610153, 1 : 1000; and anti‐GAPDH HRP‐conjugated, Antibodies‐online #ABIN398425, 1 : 3000). For HEMO and β‐catenin staining, membranes were then incubated with anti‐mouse HRP‐conjugated secondary antibody (GE Healthcare, Buc, France, 1 : 5000) for 45 min at room temperature. Protein detection was performed by using enhanced chemiluminescence reagents (Pierce™ ECL plus; Thermo Fisher Scientific) and ImageQuant LAS400 camera system (GE Healthcare).

### 2.10 Statistical analyses

All statistical analyses were performed using prism Software, v6.2 (GraphPad, https://www.graphpad.com/scientific‐software/prism/).

## 3 Results

### 3.1 *HEMO* activation is detected by *in silico* analysis in solid tumors

**Fig. 1:**
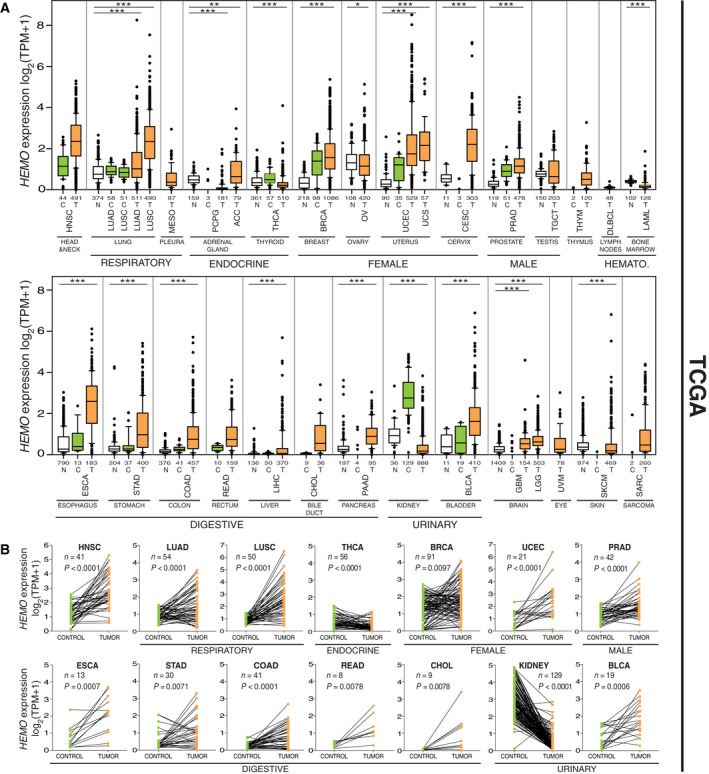
*HEMO* is overexpressed in primary solid tumors. (A) Boxplots of normalized (TPM) and log_2_‐transformed expression of *HEMO* in tumor (‘T’, orange boxplots) and control (‘C’, green boxplots) adjacent tissue samples retrieved from TCGA (The Cancer Genome Atlas)‐Recount2. The names of the cohorts are given in Table 1. White boxes correspond to basal expression in normal tissues (‘N’) from GTEx (Genotype‐Tissue Expression)‐Recount2. The number of samples analyzed in each group (N, C, T) is shown on the *x* axis (see also Table S3). The *P*‐values of pairwise comparisons between each group (T, C, N) are given in Table S3 and the (N, T) *P*‐values are shown as asterisks above the boxplots: *P < 0.05; **P < 0.01; ***P < 0.001, Mann–Whitney *U*‐test. Cohorts are grouped according to body systems. The breast cohort, which consisted primarily of female samples, include 12 male samples with similar heterogenous *HEMO* expression levels (Table S4). HEMATO., hematological tumors. Data are shown as mean with 25–75th percentile range (box) and 10–90th percentile range (whiskers). Mild outliers are depicted as black dots. (B) Matched *HEMO* gene expression analysis in pairs (Control + Tumor) from TCGA (LIHC cohort not depicted due to nonsignificant *P*‐value). *n*, number of pairs in each cohort; *P*, *P*‐values (Wilcoxon matched‐pairs test).

In order to estimate *HEMO* expression in tumors, we analyzed the RNAseq‐based transcriptome of cancer samples retrieved from TCGA (The Cancer Genome Atlas) cohorts. *HEMO* expression levels observed in tumors were then compared to those of TCGA's non‐tumor adjacent tissues (‘Control’) as well as non‐diseased (‘Normal’) tissues from the GTEx (Genotype‐Tissue Expression) project (lists in Table S3). As shown in Fig. 1A, global activation of the *HEMO* gene was observed in several TCGA tumor cohorts (orange boxes), frequently associated with high heterogeneity between samples in a given cohort. Evidence of high‐expressing cases was highlighted for a series of solid tumors, as for instance the carcinomas of endometrium (UCEC). In contrast, low levels of *HEMO* expression were generally observed in GTEx normal tissues (white boxes), in agreement with our previous results based on microarray data [31]. Most of the TCGA's control adjacent tissues (green boxes) displayed *HEMO* levels similar to those of GTEx tissues, with some discrepancies in cohorts such as breast, uterus, and prostate, reflecting a possible precancerous state in corresponding adjacent TCGA tissues.

**Table 1:**
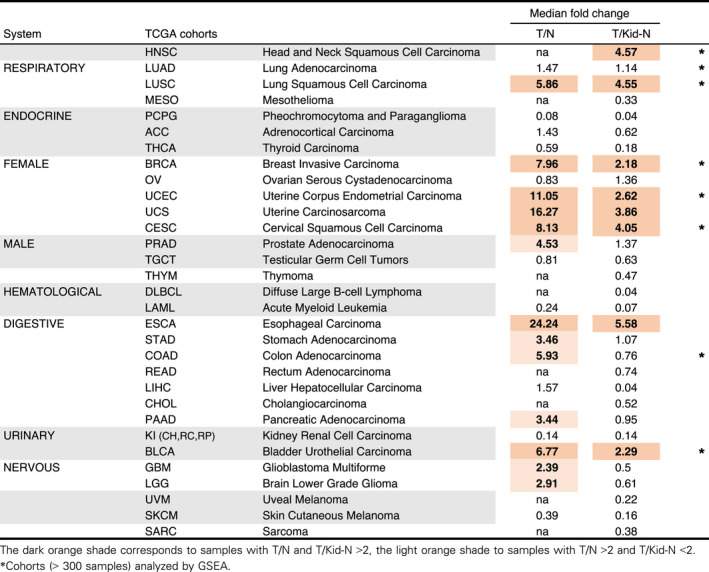
Relative expression level of *HEMO* in tumor samples compared to normal tissue samples. Median fold change (based on TPM values), between the medians of normal‐GTEx and tumor‐TCGA samples for each cohort (T/N), and between the medians of kidney normal‐GTEx and other tumor‐TCGA samples (T/Kid‐N). KI (CH, RC, RP): Kidney cohort aggregated from KICH (Kidney Chromophobe), KIRC (Kidney Renal Clear Cell Carcinoma) and KIRP (Kidney Renal Papillary Cell Carcinoma). Fold changes > 2 are in bold.

However, in kidney samples aggregated from the three cohorts KIRC, KIRP, and KICH, we could observe an unexpectedly high level of *HEMO* expression in TCGA's control compared to both GTEx and tumor samples, and this pattern was observed in the three individual kidney cohorts (Fig. S1E). A similar expression profile, although to a much lesser extent, was also noted for the thyroid (THCA) samples. The *HEMO* transcripts were not activated in ovarian serous adenocarcinoma (OV), which corresponds to the only histological subtype represented in the TCGA's ovarian cohort. This result is in line with our previous findings that demonstrated a specific upregulation of *HEMO* expression in clear cell and endometrioid ovarian adenocarcinoma but not in serous and mucinous histotypes [31]. Unlike a large proportion of solid tumors, hematological tumors (DLBCL and LAML) did not exhibit any *HEMO* activation.

Overall, to quantify *HEMO* activation in the different tumors, the fold change between the medians of normal‐GTEx samples and tumor‐TCGA samples was calculated for each individual cohort, based on TPM values (T/N median fold change in Table 1). However, because the kidney is a critical organ for survival and has a low but significant basal level of *HEMO* expression (white box), it is necessary to take this into account when evaluating the use of HEMO as a therapeutic target in non‐renal tumors. Therefore, we calculated for each cancer type, the fold change between the medians of kidney normal‐GTEx, and each tumor‐TCGA cohort (T/Kid‐N median fold change in Table 1).

Accordingly, cohorts of the lung (LUSC), breast (BRCA), uterus (UCEC, UCS), cervix (CESC), esophagus (ESCA), and bladder (BLCA) showed significant tumor activation of *HEMO*, clearly above the level of *HEMO* expression in healthy kidney (median fold changes T/N and T/Kid‐N > 2). Although the T/N value was not available in the case of the head and neck tumors (HNSC), they most likely belong to the same group. Finally, tumors of prostate (PRAD), stomach (STAD), colon (COAD)—and likely rectum (READ)—pancreas (PAAD) and at a reduced level tumors of brain (GBM, LGG) can be grouped into a second category of tumors with lower but significant *HEMO* activation (T/N > 2, T/Kid‐N < 2).

To further verify specific activation of *HEMO* in tumors, pairs of samples from the TCGA database were individually compared in representative cohorts, as illustrated in Fig. 1B. *HEMO* mRNA level was significantly enhanced in tumors compared with matched control adjacent tissues, with less pronounced effect for BRCA (breast) samples as mentioned for the boxplot. As noticed above, unexpected high values in TCGA's control samples were observed for the kidney as well as a significant decrease of expression in almost all sample pairs, a pattern similarly observed at a reduced level in the THCA (thyroid) samples.

**Fig. 2:**
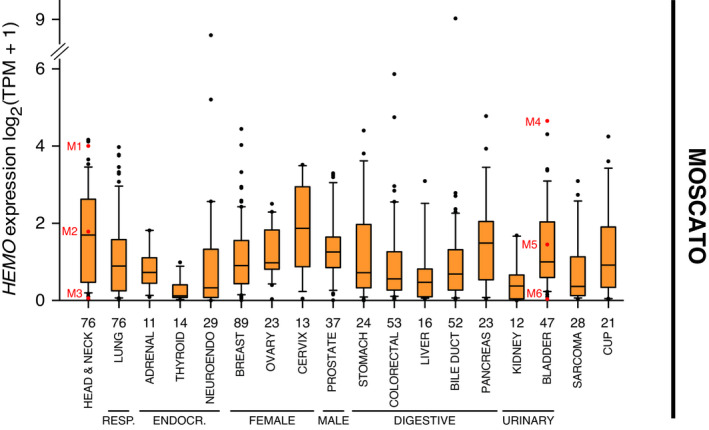
*HEMO* is overexpressed in metastatic tumors. Boxplots of normalized (TPM) and log_2_‐transformed *HEMO* expression in metastatic tumors from MOSCATO dataset (*n* = 644 samples), differently processed compared to Recount data (see Materials and methods). The organ names correspond to the localization of the primary tumor. The number of samples analyzed in each group is shown on the *x* axis. Samples marked in red (M1 to M6) are those selected for anti‐HEMO staining (Fig. 3). RESP., respiratory system; ENDOCR., endocrine system; NEUROENDO., neuro‐endocrine tumor; CUP, carcinoma of unknown primary. Data are shown as mean with 25–75th percentile range (box) and 10–90th percentile range (whiskers). Mild outliers are depicted as black dots.

Given that most of these TCGA cancer samples correspond to primary tumors (apart from the TCGA's skin ‘SKCM’ cohort), we decided to go further with the transcriptome analysis of metastatic tumors (Fig. 2). In this regard, we measured *HEMO* expression in RNAseq data retrieved from the institutional clinical trial MOSCATO (MOlecular Screening for CAncer Treatment Optimization) [34] differently processed compared to Recount data (see Materials and methods). In this study encompassing metastatic or locally advanced tumor samples, *HEMO* transcripts were also detected in a significant proportion of tumors, with similar heterogeneity within a specific tumor site. Although absolute values could not be directly compared, and despite smaller number of samples, the rank order of the cohorts based on their *HEMO* expression was on the whole conserved between analyses of primary (TCGA) and metastatic (MOSCATO) tumors. Highest median values were observed in metastatic tumors of head and neck, cervix, and pancreas, while thyroid and kidney metastatic tumors remained at low levels.

To investigate whether HEMO is a marker of cancer progression, we stratified samples by tumor stage and grade, in the TCGA cohorts where the increase of *HEMO* expression in tumors was the highest compared to normal tissues (Fig. S2). Only a few significant associations were found between *HEMO* expression and each parameter, as in the HNSC (Head and Neck) and PAAD (pancreas) cohorts in which *HEMO* expression was positively correlated with high tumor grades (G2‐G3 and G3, respectively). Further analysis of histological types indicated that in the esophagus tumors, *HEMO* was preferentially expressed in the squamous cell carcinoma subtype (SCC) compared to the adenocarcinoma subtype (ADC). Of note, this difference was also observed for the lung tumors, between the lung squamous cell carcinoma (LUSC) and lung adenocarcinoma (LUAD) cohorts in Fig. 1A. Finally, stratification of breast tumor samples by their molecular signature revealed that *HEMO* was poorly activated in the Her2+ subgroup.

### 3.2 Tumor expression of other *HERV‐env* genes

Next, we wanted to investigate if other well‐known endogenous retroviral envelope genes were also overexpressed in tumor condition. Among the still coding envelope genes of the human genome [37, 38], four have specific characterized transcripts and a reliable Ensembl annotation (Table S5). We analyzed the *syncytin‐1* (*ERVW‐1*) and *syncytin‐2* (*ERVFRD‐1*) transcripts (which are known to encode identified placental proteins), *ERVV‐2* (a placental specific transcript), and the more ubiquitous *ERV3‐1* gene (encoding a putative soluble envelope protein).

As shown in Fig. S1, and by contrast with *HEMO*, the *syncytin‐1* and *ERVV‐2* envelope coding sequences displayed almost undetectable expression and were poorly activated in tumor conditions. High level of *ERVW‐1* expression was detected in normal testis, but was not specifically enhanced in testis cancer (*P*‐value in Table S3). In the case of *ERVV‐2*, we could observe a few samples with high level of expression, notably in renal tumors (see also Fig. S1E), which need further investigations to confirm detection at the protein level.

Strikingly, the *syncytin‐2* (*ERVFRD‐1*) sequence, which had a basal level of expression in most normal tissues, was slightly repressed in control adjacent tissues, and even more repressed in tumor conditions, except for the kidney, where we could observe faint *ERVFRD‐1* activation in control adjacent tissues, as for *HEMO*. This was confirmed in the three individual kidney cohorts (Fig. S1E), but was not a general case for the other *HERV‐env* sequences.

Nevertheless, we noticed that *syncytin‐1* (*ERVW‐1*) and *syncytin‐2* (*ERVFRD‐1*) were activated in acute myeloid leukemia (LAML).

Finally, *ERV3‐1* did not clearly appear as a tumor‐specific transcript since most of the cohorts displayed similar expression in normal tissues, control, and tumor samples, with sporadic activation or inactivation of the sequence (Fig. S1D). Still, it can be noted that its highest median value is observed in the LAML cohort.

Analysis of other full‐length envelope coding genes could not be performed, mainly due to the multigenic nature of some HERV family, such as in the HERV‐K(HML‐2) family [39], preventing the clear identification of the few coding copies.

### 3.3 HEMO is detected at the protein level

**Fig. 3:**
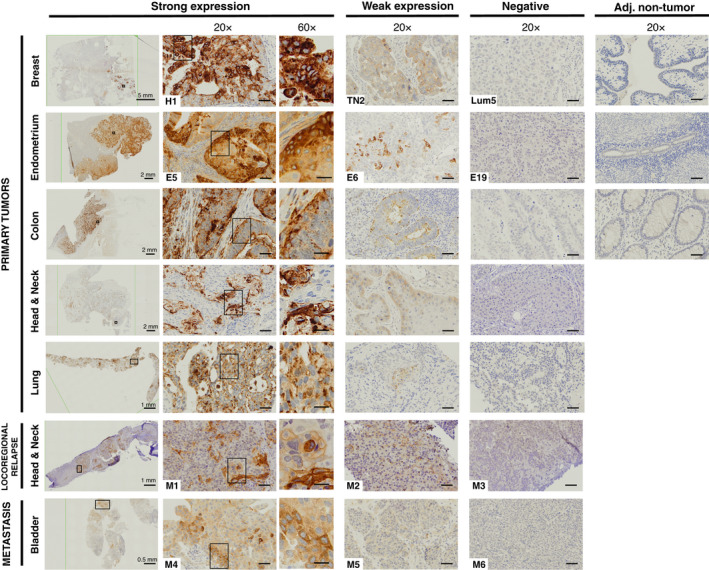
HEMO protein is detected in various tumor tissues. Examples are shown of strong, weak, and absent HEMO expression in cancer samples and of no detection in adjacent non‐tumor samples (staining with anti‐HEMO 2F7 mAb). Left column (0.4–2×): scale bar indicated in mm, black frame defined localization of 20× view. 20× columns: scale bars correspond to 50 μm, black frame defined localization of 60× view. 60× column: scale bars correspond to 20 μm. Numbers displayed on breast (H1, TN2, Lum5) and endometrium (E5, E6, E19) samples correspond to numbers used in Fig. 4. Numbers displayed on locoregional relapse samples and metastatic samples (MOSCATO M1 to M6) correspond to those marked in red in Fig. 2.

To confirm the *in silico* data, pan‐cancer immunohistochemistry analyses were performed on primary tumor FFPE samples from Gustave Roussy (*n* = 126, in Table 2) with a specific anti‐HEMO monoclonal antibody [31]. Evidence of weak to strong staining was found in different tumor tissues, while on the contrary, some other tumors were deprived of HEMO protein, as shown in a representative sampling of tumor tissues (Fig. 3). In our samples, the HEMO protein was not detectable in available adjacent non‐tumor tissues. When present, staining was generally scattered within the tumor area. At the cell level, HEMO localized to the cytoplasmic and membrane compartments, as expected for a membrane protein (see also staining in cancer cell lines, Fig. S3C) and in agreement with our previous results [31].

**Table 2:** Distribution of *HEMO* expression in pan‐cancer cohort as measured by immunohistochemistry. HEMO expression was classified as strong, weak, or negative according to the strength and the prevalence of the staining within the sample. Samples include primary and metastatic (M: number in brackets) tumors. ‘Organ’ corresponds to the primary localization.

System	Organ	Tumor type	Strong	Weak	Negative
	Head and neck	Squamous carcinoma	9	9	17
RESPIRATORY	Lung	Adenocarcinoma	7		2 (M = 1)
		Squamous carcinoma	1	2	1
FEMALE	Breast	Carcinoma	3	7 (M = 3)	13 (M = 3)
	Endometrium	Adenocarcinoma	3	2	6
	Cervix	Squamous carcinoma			3
MALE	Prostate	Adenocarcinoma			3
DIGESTIVE	Esophagus	Adenocarcinoma	2	1	1
		Squamous carcinoma	1	0	1
	Stomach	Adenocarcinoma	1	1	2
	Colon	Adenocarcinoma	4 (M = 1)	4	2
	Rectum	Adenocarcinoma	6 (M = 1)	1	
	Skin	Melanoma	1 (M = 1)	1 (M = 1)	9 (M = 9)
% of Total (126 cases)			30%	22%	48%

As reported in Table 2, strong HEMO staining was detected in 30% of this limited pan‐cancer cohort, with most of the cases found in head and neck squamous carcinoma (9/32), lung carcinoma (8/13), breast carcinoma (3/23), endometrium adenocarcinoma (3/11), esophagus (3/6), colon adenocarcinoma (4/10), and rectum adenocarcinoma (6/7), thereby confirming the tendency predicted by TCGA data.

In addition, in order to precisely compare RNAseq results and degree of protein detection in the same sample, tumors included in the MOSCATO cohort were also stained with anti‐HEMO antibody (Fig. 3, bottom). As shown for locoregional relapse of head and neck tumors and bladder metastases, we found a clear correlation between the amount of HEMO protein and the expression levels estimated from transcriptomic data of Fig. 2 (M1–M6 samples).

**Fig. 4:**
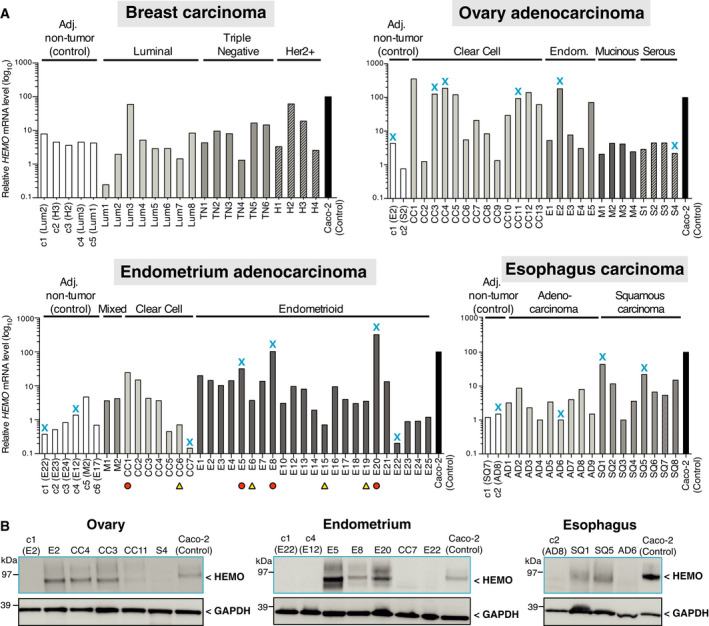
Detection of *HEMO* transcripts and corresponding protein in breast, ovary, endometrium and esophagus tumor samples. (A) Expression of *HEMO* measured by RT‐qPCR. Amount of mRNA was normalized to *RPLP0* transcript level. Caco‐2 mRNA was used as positive control for *HEMO* expression. Bar plots represent mean expression of two independent technical replicates. Blue cross marks indicate samples analyzed by western blot in part B. Orange circles and yellow triangles, respectively, correspond to HEMO^Strong^ and HEMO^Weak/Neg^ samples selected for Fig. 6. Adj., adjacent; Endom., endometrioid. (B) Detection of glycosylated full‐length protein HEMO in corresponding lysates of ovary (left), endometrium (middle) and esophagus (right) tumors. Caco‐2 lysate was used as positive control for HEMO molecular weight. GAPDH was measured as a control of cell lysate protein loading. Results shown are representative of three independent technical replicates.

To get further into the unambiguous HEMO identification, we analyzed its expression in frozen tumor samples of breast, ovary, endometrium, and esophagus by RT‐qPCR and western blot analysis (Fig. 4). As for the TCGA results, control adjacent tissues displayed some slight variation in *HEMO* transcription levels. Heterogeneity with multiple high values of *HEMO* expression was observed in tumor samples. Consistently with these transcription results, we clearly detected by western blot a band with an apparent molecular mass similar to what is expected for the glycosylated full‐length HEMO protein in corresponding tumor lysates. Such band was not observed in *HEMO* negative tumors or surrounding non‐tumor tissues (Fig. 4B). Taken together, these results support the highly specific expression of the endogenous retroviral HEMO envelope in solid tumors.

### 3.4 *HEMO* upregulation is linked to Wnt/β‐catenin signaling in endometrial tumors

To further characterize endometrial tumors expressing *HEMO* and identify potential co‐expression signatures in these tumors, we performed differential gene expression analysis between HEMO^High^ and HEMO^Low^ tumors from TCGA's UCEC cohort (Table S6). Patients were separated into ‘High’ or ‘Low’ *HEMO* expression groups, using 90th and 10th percentiles as cutoffs (size: 53 patients in each group). Next, Gene Set Enrichment Analysis (GSEA) was performed using REACTOME and Gene Ontology (GO) Biological Process (BP) databases (Table S7).

**Fig. 5:**
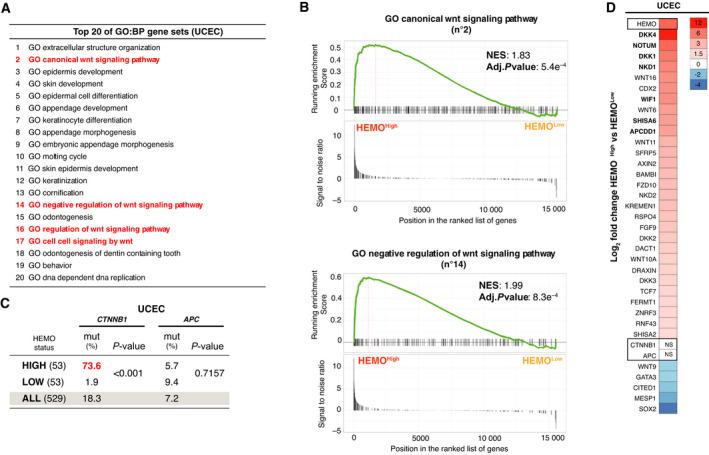
*HEMO* upregulation is associated with an active Wnt/β‐catenin pathway in UCEC cohort. (A) Top 20 GO (Gene Ontology) BP (Biological Process) gene sets with the highest significance in UCEC cohort, extracted from Table S7. In red: Wnt signaling pathways. (B) GSEA enrichment plots showing ‘GO canonical Wnt signaling pathway’ and ‘GO negative regulation of Wnt signaling pathway’ enriched signatures between UCEC HEMO^High^ vs HEMO^Low^ tumors. NES: Normalized Enrichment Score. (C) Mutational status of *CTNNB1* and *APC* in UCEC HEMO^High^ and HEMO^Low^ tumors, 53 cases of each (*P*‐value, Fisher's exact test). For comparison, mutational status of the total UCEC cohort is indicated. (D) Heatmap for the significant Wnt‐related genes differentially expressed between HEMO^High^ and HEMO^Low^ tumors. Color gradation is representative of Log_2_ fold change. The differential level of *HEMO* expression is also indicated at the top of the heatmap (LogFC = 7). For all depicted genes, adjusted *P*‐value is < 0.01 (except for *APC* and *CTNNB1*, NS: nonsignificant). In bold, common upregulated genes found in COAD cohort (Fig. S4B).

It revealed that among enriched gene sets, several pathways were related to Wnt/β‐catenin signaling, (see also results in paragraph 3.5), with four of them (marked with an asterisk) belonging to the top 20 most significant GO gene sets, listed in Fig. 5A. Enrichment plots of ‘GO canonical Wnt signaling pathway’ (number 2) and ‘GO negative regulation of Wnt signaling pathway’ (number 14) are illustrated in Fig. 5B, with positive Normalized Enrichment Score (NES) and robust adjusted *P*‐value (< 0.001).

**Table 3:** Wnt‐related pathways are enriched in HEMO^High^ tumors in TCGA cohorts. For each TCGA cohort, normalized enrichment score (NES, bold) and adjusted *P*‐value (in brackets) of GSEA (performed on Gene Ontology Biological Process and REACTOME databases) are given when the Wnt‐related pathway is significantly enriched. RESP., respiratory; Green: terms illustrated on enrichment plots of Fig. 5B and Fig. S4A.

	RESP.	FEMALE				DIGESTIVE	URINARY
	LUSC	BRCA		UCEC	CESC	COAD	BLCA
GO canonical Wnt signaling pathway*	**1.42** (0.017)	**1.45** (0.042)		**1.83** (5.4e‐4)	**1.56** (0.011)		
GO negative regulation of Wnt signaling pathway*				**1.99** (8.3e‐4)	**1.63** (0.019)		
GO regulation of Wnt signaling pathway*	**1.41** (0.012)	**1.62** (2.3e‐3)		**1.77** (1.0e‐3)	**1.59** (3.3e‐3)		
GO cell signaling by Wnt*		**1.52** (4.7e‐3)		**1.70** (1.0e‐3)	**1.48** (6.0e‐3)		
GO negative regulation of canonical Wnt signaling pathway				**1.90** (2.2e‐3)			
GO beta catenin TCF complex assembly							**1.87** (0.019)
REACTOME negative regulation of TCF dependent signaling by Wnt ligand antagonists				**1.87** (0.015)			
REACTOME signaling by Wnt in cancer				**2.13** (6.3e‐4)			
REACTOME Wnt ligand biogenesis and trafficking				**1.79** (0.047)			
REACTOME formation of the beta catenin TCF transactivating complex						**2.00** (2.0e‐3)	
REACTOME TCF dependent signaling in response to Wnt						**1.64** (0.013)	
REACTOME signaling by Wnt						**1.48** (0.036)	

* Terms belonging to the top 20 list of Fig. 5A.

Such associations with active Wnt/β‐catenin signaling led us to analyze in each subgroup of UCEC tumors (HEMO^High^ and HEMO^Low^) the mutational status (Fig. 5C) and the expression level (Fig. 5D) of two key genes of this pathway, namely *CTNNB1* (β‐catenin coding gene) and *APC* (adenomatous polyposis coli gene). Alterations in these genes are indeed frequently found in endometrial tumors and lead to a constitutive activation of the Wnt/β‐catenin pathway [30]. In the UCEC TCGA cohort, 18.3% of the samples are mutated in *CTNNB1*, and 7.2% in *APC* gene (Fig. 5C). Importantly and convincingly, we found that 73.6% (39 of 53) of HEMO^High^ versus 1.9% (1 of 53) of HEMO^Low^ tumors harbored stabilizing mutations in *CTNNB1* (*P* < 0.0001), known to drive a drastic activation of the Wnt/β‐catenin pathway, which was consistent with the GSEA results. Conversely, no association with *APC* mutations was observed (Fig. 5C). None of these two genes were differentially expressed (Fig. 5D). A heatmap of the relative changes in Wnt/β‐catenin signaling‐related gene expression (Fig. 5D) showed that most genes involved in this pathway were upregulated in HEMO^High^ tumors with a noticeable upregulation of *DKK4* (LogFC = 12), a member of the Wnt inhibitor DKK family [40].

**Fig. 6:**
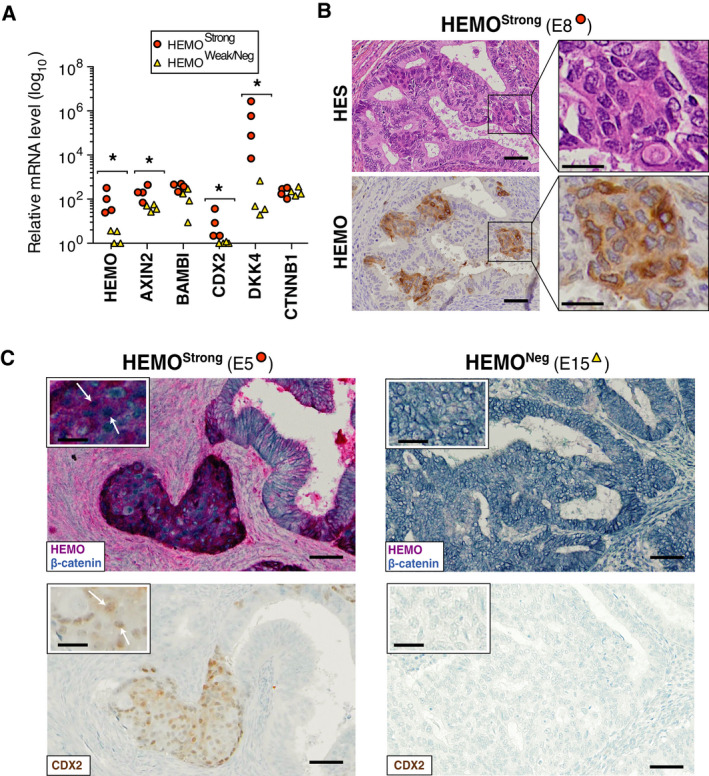
HEMO is detected in morular metaplasia of endometrium adenocarcinoma displaying activation of the Wnt/β‐catenin pathway. (A) Transcript levels of representative Wnt‐related genes overexpressed in HEMO^High^ UCEC tumors measured by RT‐qPCR on endometrium adenocarcinoma samples, classified as HEMO^Strong^ (*n* = 4 samples) or HEMO^Weak/Neg^ (*n* = 4 samples) according to their *HEMO* expression level, previously estimated by RT‐qPCR in Fig. 4. Amount of mRNA was normalized to *RPLP0* expression level (*P < 0.05, Mann–Whitney test). (B) HES and immunohistochemical detection of HEMO (2F7 mAb) in morular metaplasia of endometrium adenocarcinoma. Left, magnification: 20×, scale bar: 50 μm; right, magnification: 60×, scale bar: 20 μm. (C) Co‐staining of HEMO (red) and β‐catenin (blue) in examples of HEMO^Strong^ (left) and HEMO^Neg^ (right) endometrial adenocarcinoma (top) and CDX2 nuclear staining (bottom). Morular foci displayed nuclear β‐catenin and CDX2 staining (as shown by white arrows). Of note, nuclear β‐catenin staining was detected in 3 out of the 4 HEMO^Strong^ samples (1 undetermined) and was not detected in the 4 HEMO^Weak/Neg^ samples. Magnification: 20×, scale bar: 50 μm; insert, magnification: 40×, scale bar: 20 μm. Samples are identified as in Fig. 4.

Following these findings, the expression of a series of genes upregulated in the UCEC HEMO^High^ versus HEMO^Low^ tumors was examined by RT‐qPCR in our endometrium tumor samples, selected among those of Fig. 4 with corresponding HEMO ‘Strong’ and ‘Weak/Neg’ expression level (indicated by orange circles and yellow triangles). As shown in Fig. 6A, *AXIN2*, *DKK4,* and *CDX2* were found to be significantly upregulated in HEMO^Strong^ compared to HEMO^Weak/Neg^ tumors. Given that these genes are considered as negative regulators of the Wnt/β‐catenin pathway, their overexpression could indicate a negative response developed to counteract abnormal activation of the pathway, as already described [41]. Immunohistochemistry analyses by HEMO and β‐catenin co‐staining were performed on the endometrial tumor samples (Fig. 6C). In HEMO^Strong^ tumors, HEMO colocalized in specific tumor cells in which nuclear β‐catenin was observed (white arrow in sample E5), whereas in *HEMO* negative cells, either in the same sample or in HEMO^Weak/Neg^ tumors (sample E15), the β‐catenin was mainly localized to the cell membrane.

Nuclear localization of β‐catenin reflects its stabilization induced by mutations of either *CTNNB1* or *APC* and results in activation of the signaling pathway, therefore without any up‐ or downregulation of *CTNNB1* expression, as observed in Fig. 6A. *CTNNB1* mutations and subsequent nuclear accumulation of β‐catenin have been already reported in endometrial adenocarcinoma and in particular in foci of morular metaplasia [42, 43]. Strikingly, we noticed that HEMO prominent areas in endometrium adenocarcinoma matched with morules clearly identified in HES (Fig. 6B) and positively stained with CDX2 (Fig. 6C), known as being expressed in these typical elements [44].

Taken together, these results clearly demonstrate that HEMO is preferentially found in tumors with an active Wnt/β‐catenin pathway. Therefore, in addition to cell morphological modifications, this pathway could participate in activation of *HEMO* in tumors.

### 3.5 *HEMO* upregulation and Wnt/β‐catenin signaling in other tumors

Next, we sought to determine if HEMO^High^ tumors from TCGA cohorts other than UCEC (marked with an asterisk in Table 1) displayed a similar relationship with the Wnt/β‐catenin pathway. The Gene Set Enrichment Analyses (Tables [Link], [Link]) performed on HNSC, LUAD, LUSC, BRCA, CESC, COAD, and BLCA cohorts showed that signatures related to the Wnt/β‐catenin signaling pathway were also enriched in BRCA, CESC, and COAD and to a lesser extent in LUSC and BLCA cohorts (Table 3). Of note, no GO nor REACTOME gene sets were found to be enriched in the HNSC cohort under our conditions of GSEA (adj. *P*‐value < 0.05).

*APC* and *CTNNB1* mutations were then screened in HEMO^High^ and HEMO^Low^ groups of these TCGA cohorts (Table S8). Particularly in the COAD cohort, the prevalence of *APC* mutations was significantly higher (*P* = 0.0016) in HEMO^High^ compared with HEMO^Low^ group (67.4% vs 32.6%). Thus, this increase could be linked to enrichment in the Wnt‐related pathways reported in Table 3. As shown in the heatmap of Fig. S4B, and similarly to UCEC, COAD HEMO^High^ tumors displayed absence of differential expression of *CTNNB1* and *APC*, as well as an upregulation of members of the *DKK* gene family and *NOTUM*. However, in the COAD cohort, GSEA revealed enrichment in REACTOME pathways, such as ‘formation of the beta catenin TCF transactivating complex’ or ‘TCF dependent signaling in response to Wnt’ (Fig. S4A). These were not shared by the UCEC cohort, mainly due to some differences found in their profiles of upregulated Wnt/β‐catenin signaling‐related genes observed in the two heatmaps.

Overall, these results suggest that *HEMO* activation in tumor condition and, in particular, in endometrial tumors may involve Wnt/β‐catenin signaling.

### 3.6 *HEMO* expression is modulated by the Wnt pathway in cell models

To specifically address whether regulation of *HEMO* expression is modulated by the Wnt/β‐catenin signaling pathway in tumors, inhibition of this pathway was performed on cell models. Among six cell lines which endogenously express *HEMO* (NCIH520, HCC827, OVMANA, Caco‐2, SW480, and HCT116), the lung squamous cell carcinoma NCIH520 and colorectal adenocarcinoma Caco‐2 cell lines were selected since they showed the highest *HEMO* expression levels (Fig. S3). Besides, Caco‐2 cells are known to harbor somatic mutations in both *CTNNB1* and *APC* [45], thus constituting an example of cells under an active Wnt/β‐catenin pathway. Conversely, the NCIH520 model does not exhibit such mutations.

**Fig. 7:**
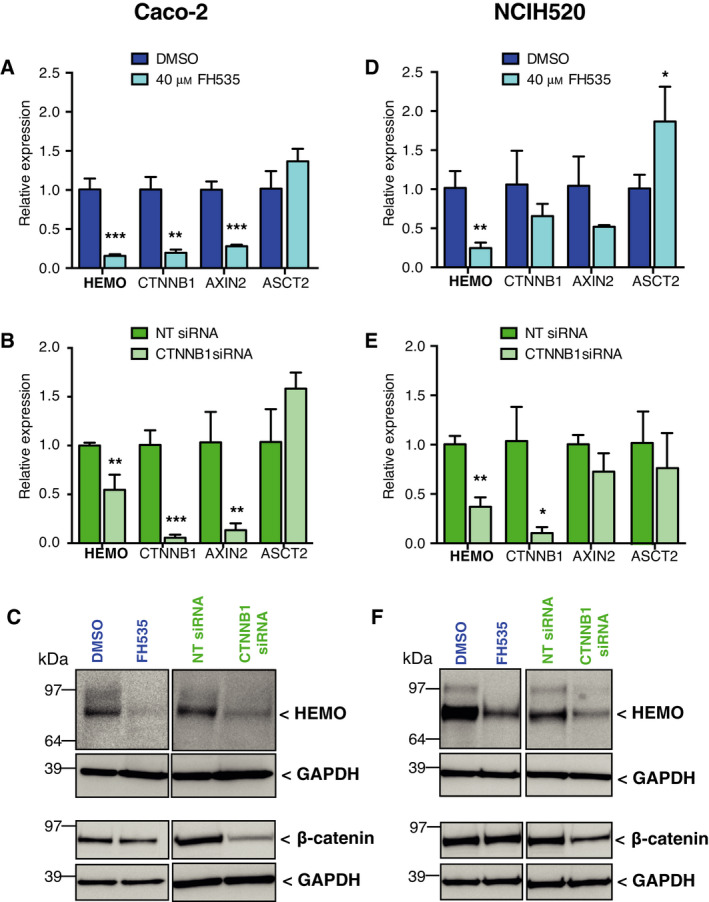
*HEMO* expression decreases after Wnt/β‐catenin inhibition in Caco‐2 (A–C) and NCIH520 (D–F) cells. Inhibition was performed with 40 μm of FH535 (A: Caco‐2, 72 h; D: NCIH520, 24 h) or by *CTNNB1* silencing with siRNAs (B, E). Amount of mRNAs was normalized to housekeeping gene *RPLP0* transcript level. Bar plots represent mean relative expression of three independent RT‐qPCR experiments ± SD. (*P < 0.05; **P < 0.01; ***P < 0.001, unpaired Student's *t*‐test). Corresponding HEMO and β‐catenin protein amount was assessed by western blot (C, F). GAPDH was measured as a control of cell lysate protein loading. NT: Non‐targeting; ASCT2: neutral amino acid transporter.

First, cells were treated with FH535, a chemical inhibitor of transcription mediated by the TCF/β‐catenin complex [46]. Expression of *HEMO*, *CTNNB1,* and the Wnt/β‐catenin target gene *AXIN2*, was measured by RT‐qPCR (Fig. 7). We found that treatment by FH535 significantly decreased *HEMO* transcript level in Caco‐2 and NCIH520 cells while expression of *ASCT2* was not reduced (Fig. 7A,D). This result was further confirmed by western blot analysis with a strong reduction of the corresponding HEMO protein in both cell lines (Fig. 7C,F, left). In contrast, transcription levels of *CTNNB1* and its target gene *AXIN2* decreased with varying degrees of downregulation in the two cell lines. Whether it is associated with diverse toxicity effects of the FH535 drug on carcinoma cell lines [46] or with differential genetic background of these two cell lines needs to be further investigated.

Using a more specific approach, the same cell lines were then transfected with a pool of small interfering RNAs directed against *CTNNB1*. The efficacy of this siRNA was first validated, as shown by the significant reduction of *CTNNB1* mRNA levels (Fig. 7B,E) and of the corresponding β‐catenin protein (Fig. 7C,F, right). In that condition, a significant downregulation of *HEMO* transcription was observed in the Caco‐2 and NCIH520 cell lines, which resulted into an extensive decrease of the HEMO protein. Concerning *AXIN2*, responses to inhibition by siRNA were consistent with those observed with the drug. Overall, these experiments, carried out in two independent cell lines, provided evidence that the Wnt/β‐catenin signaling pathway participates in the regulation of *HEMO* expression.

### 3.7 *HEMO* activation is associated with keratinization and immune signatures

Besides identification of Wnt/β‐catenin signaling as a putative regulator of *HEMO* expression, GSEA performed on the TCGA cohorts (enriched pathways listed in Table S7) led us to identify other gene expression patterns associated with *HEMO* activation.

**Fig. 8:**
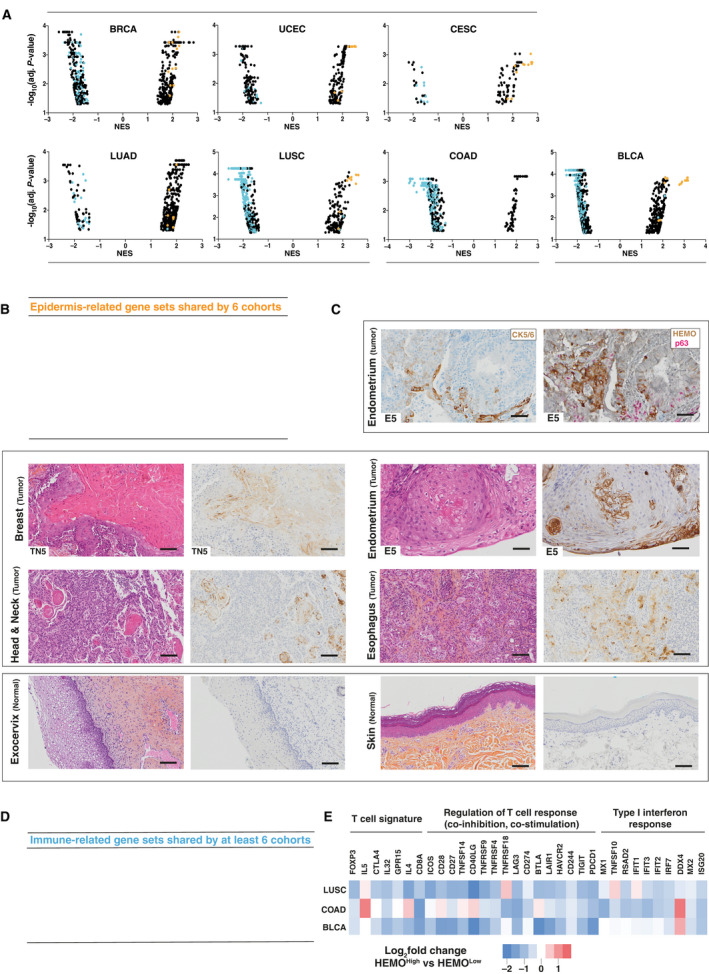
*HEMO* activation in tumors is linked to epidermis and immune‐related signatures. (A) GSEA (Gene Ontology Biological Process and REACTOME) pathway distribution for HEMO^High^ vs HEMO^Low^ tumors in TCGA cohorts. Of note, for each cohort, these two groups displayed similar tumor cellularity. Immune response‐ and epidermis‐related gene sets are, respectively, demarcated as blue and orange dots. NES: Normalized Enrichment Score. (B) List of epidermis‐related gene sets shared by six cohorts (absence of such gene sets in the COAD cohort). (C) Top: Endometrium adenocarcinoma sample stained with CK5/6 (left) or HEMO (brown) and p63 (pink) (right), magnification: 15×, scale bar: 100 μm. Middle: pairs of HES and anti‐HEMO staining in differentiated squamous tumors of breast, endometrium, Head and Neck with peculiar localization of HEMO in squamous pearls and esophagus. Bottom: pairs of HES and anti‐HEMO staining in normal tissue of exocervix and skin. Magnification: 10×, scale bar: 100 μm. When specified, numbers correspond to samples analyzed in Fig. 4. (D) List of immune‐related gene sets shared by 6 or 7 cohorts. (E) Heatmap displaying differential expression between HEMO^High^ and HEMO^Low^ tumors, of immune‐related genes belonging to ‘T‐cell (Treg and CD8)’, ‘Regulation of immune response’ and ‘Type I interferon response’ signatures established by [47]. *PDCD1* was also added on this figure. Color gradation is representative of Log_2_ fold change.

Remarkably, we could identify that epidermis development or keratinization gene sets were strongly enriched in HEMO^High^ tumors compared to HEMO^Low^ tumors in the BRCA, UCEC, CESC, LUSC, and BLCA cohorts (as already depicted in the top 20 list for the UCEC cohort in Fig. 5A). Of note, absence of these gene sets was observed in the COAD cohort. As shown in Fig. 8A illustrating the distribution of all the GSEA gene sets for the seven cohorts, these epidermis‐related pathways (orange dots), belonged to the most significantly enriched pathways (Adj. *P*‐value < 0.01, NES > 2). The pathways common to at least six cohorts are listed in Fig. 8B. To confirm these bioinformatics data, we performed immunohistochemistry assays (Fig. 8C). The HEMO protein was detected in squamous and keratinizing nests of endometrium adenocarcinoma, highlighted by CK5/6 and p63‐positive staining (Fig. 8C, top). Comparison of HEMO‐positive regions with corresponding HES unveiled that this protein was frequently expressed in areas of squamous differentiation, sometimes associated with abnormal keratinization, as shown for tumors of breast, endometrium, head and neck, or esophagus tumors (Fig. 8C, middle). In contrast, HEMO was undetectable in normal squamous tissue such as exocervix, or in squamous and keratinized tissue such as skin (Fig. 8C, bottom). This observation ruled out possible non‐specific staining on these tissues, with high content of keratins in the case of skin.

Lastly, GSEA results revealed a substantial depletion of immune‐related gene sets in HEMO^High^ tumors of several TCGA cohorts (Fig. 8A, blue dots) with numerous pathways reaching very high significance (Adj. *P*‐value < 0.001, NES < −2). Interestingly, pathways belonging to both innate and adaptive immune responses were commonly under‐represented in HEMO^High^ groups of several TCGA cohorts (Fig. 8D).

Negative association with these immune signatures was particularly noticeable in LUSC, COAD, and BLCA cohorts as shown in their top 10 most significant GO:BP and REACTOME gene sets (Table S9). Indeed, a heatmap of representative genes of immune features such as ‘T cell’, ‘Regulation of T‐cell response’, and ‘Type I interferon response’ established by [47] mainly showed their underexpression in HEMO^High^ tumors (Fig. 8E).

## 4 Discussion

Altogether, the presented results indicate that the endogenous retrovirus‐derived *HEMO* envelope gene is specifically upregulated in solid primary tumors. Indeed, demonstration of the concordance between *HEMO* transcriptional activation and detection of the corresponding protein in a series of tumor tissues (by immunohistochemistry and clear identification of the protein by western blot) allowed us to validate the relevance of measuring *HEMO* expression via large scale high throughput RNAseq data.

These analyses revealed that *HEMO* is highly activated in head and neck, lung, breast, endometrium, cervix, esophagus, and bladder tumors, whereas it is faintly or not detected in normal tissues. Activation was also observed, although to a lower level, in cohorts such as prostate, stomach, colorectal, pancreatic, or brain tumors.

Furthermore, in cohorts such as breast, uterus, or prostate, we noticed, between normal and tumor conditions, an intermediate level of activation in the control adjacent tissues, suggesting a possible precancerous state or local inflammatory response [48].

However, a special case was the renal tumors, where both a strong activation in control adjacent tissues and a very clear decrease in expression in tumor tissues were observed in the kidney cohorts. This unexpected level of *HEMO* expression in tumor adjacent tissues (highest median value of all tumor samples in Fig. 1) could not simply represent a precancerous intermediate state. The kidney is a vital organ, where tissue damage reactivates specific pathways to repair renal function, in particular the Wnt/β‐catenin signaling pathway usually involved in nephron morphogenesis during embryogenesis [49, 50]. In tumor conditions, control adjacent tissues of the kidney could be engaged in active regenerative processes to compensate for the destruction of renal parenchyma, with consecutive *HEMO* activation dependent on this pathway. On the other side, the *HEMO* downregulation observed in the renal tumor cells could result from specific epigenetic modifications or from tumor amplification of renal cells not expressing *HEMO*. Indeed, single‐cell RNAseq analyses of normal tissue of the Human Protein Atlas project (https://proteinatlas.org) showed preferential *HEMO* (*ERVMER34‐1*) expression in collecting duct cells. Corresponding tumors represent only 1% of the renal cell carcinomas [51] and are not present in the TCGA consortium.

In addition, we showed that *HEMO* expression is maintained in metastatic tumors and is associated with high tumor grade in head and neck tumors and possibly in the smaller pancreatic cohort. Interestingly, it was recently reported that *HEMO* (*ERVMER34‐1*) belongs to a gene signature predicting recurrence of colon adenocarcinoma based on the comparison of mRNAs, lincRNA, and miRNA transcriptomic profiles of recurrent and non‐recurrent tumors [52]. These results unveil its putative interest as a prognostic factor.

Furthermore, in the cohorts analyzed in this study, *HEMO*'s activation in solid tumors made this retroviral envelope gene stand out from others such as *syncytin‐1* (*ERVW‐1*) and *ERVV‐2* for which we mainly found low expression, from *syncytin‐2* (*ERVFRD‐1*) usually inactivated in tumor samples, and from *ERV3‐1* which mainly shows non‐tumor‐specific activation. However, since *syncytins* and *ERVV‐2* sequences are mostly undetectable in normal tissues, their occasional activation in some tumors, as well as the *ERVW‐1* expression in testicular cells, could be of interest for specific targeted therapy. In contrast to *HEMO*, *syncytin‐1* [53] and *syncytin‐2* were specifically increased in hematological LAML tumors.

Characterization of co‐expression signature associated with *HEMO* activation in TCGA datasets led us to hypothesize that its expression could be linked to the Wnt/β‐catenin pathway, in particular in endometrium tumors, where we found a clear association between the presence of *CTNNB1* mutations and a high level of *HEMO* expression (73.6%). Altered Wnt/β‐catenin signaling, as a result of gene alterations such as those found in *APC* or in *CTNNB1* (β‐catenin), has been reported to drive tumorigenesis in numerous tumors including endometrial cancer [30]. For patients with low‐grade and early‐stage endometrioid endometrial tumors, these mutations have been associated with a higher risk of recurrence [54, 55]. From a histological standpoint, constitutive activation of the Wnt/β‐catenin pathway with abnormal nuclear accumulation of β‐catenin is preferentially observed in morules of endometrial carcinomas [42]. In our endometrium tumor samples, colocalization of HEMO and nuclear β‐catenin in specific morular metaplasia therefore confirmed the initial hypothesis indicating a close link between the Wnt/β‐catenin pathway and *HEMO* activation. Moreover, the *in vitro* inhibition of this pathway clearly provided evidence that Wnt/β‐catenin signaling is involved in *HEMO* regulation. Data from the ENCODE project supported the hypothesis that the *HEMO* promoter could be bound by TCF/β‐catenin partners or by transcription factors of the Wnt signaling pathway [56]. Chromatin‐immunoprecipitation assays in human cell lines having high levels of *HEMO* expression or targeted CRISPR experiments to modify the *HEMO* promoter or knock‐out specific members of the Wnt signaling pathway will help to establish whether *HEMO* can be considered as a *bona fide* downstream direct or indirect target of this pathway.

**Fig. 9:**
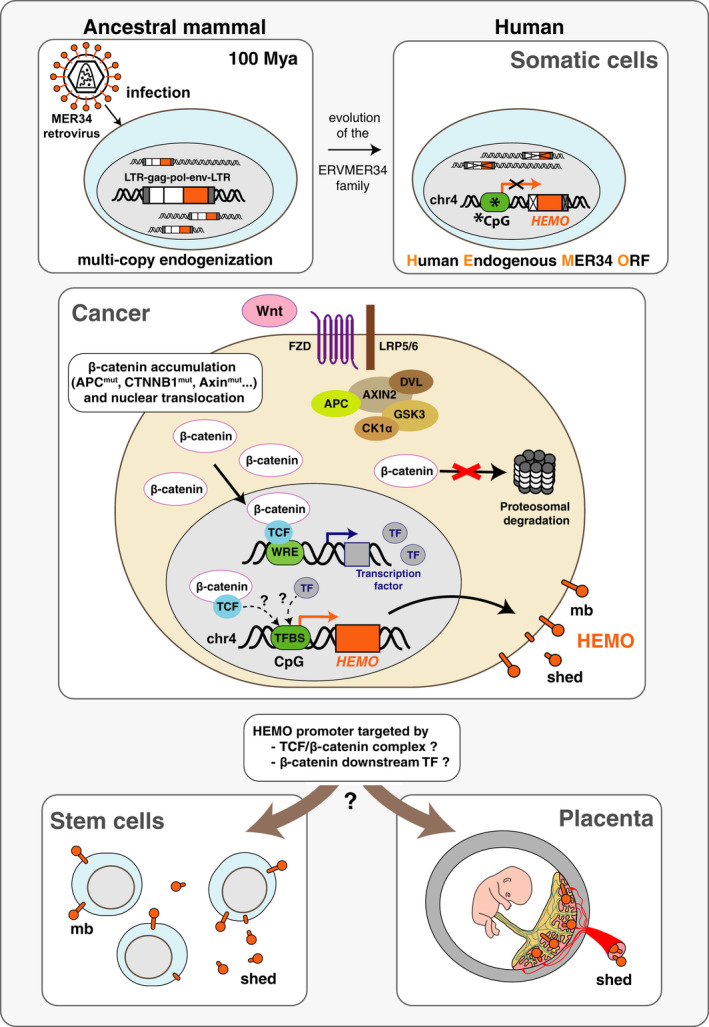
Model for the regulation of *HEMO* expression in cancer. A ‘MER34 retrovirus’ was endogenized in an ancestral mammal genome about 100 mya, as a multi‐copy ERV family. Following genetic evolution, only one *env* ORF remains present in human genome on chromosome 4, having lost its 5′ LTR and being transcribed from a cellular CpG‐rich promoter. This sequence was previously described as *HEMO* (Human Endogenous MER34 ORF) [31]. Methylation of its promoter could participate in the *HEMO* silencing in somatic cells (*CpG methylation). In cancer, besides epigenetic modifications, such as hypomethylation of the *HEMO* promoter, we propose that the Wnt/β‐catenin pathway is an upstream regulator of *HEMO* expression. Activation of this signaling pathway, due to genetic or epigenetic alterations, is frequently observed in tumors and results in the accumulation of β‐catenin and its subsequent translocation to the nucleus of the tumor cell. Once in the nuclear compartment, β‐catenin associates with TCF to form a complex that binds WREs (‘Wnt Responsive Elements’) and thus activates transcription of numerous target genes. Among them, transcription factors could in turn activate *HEMO* transcription. Alternatively, *HEMO* could directly be activated by the β‐catenin/TCF complex. These mechanisms could also drive *HEMO* activation in stem cells and placenta where this gene is expressed as membrane and shed HEMO proteins [31]. mb, membrane; LTR, long terminal repeat; TF, transcription factor, TFBS, transcription factor binding site.

Interestingly, it has been previously shown that *HEMO* is expressed in placenta and embryonic stem cells (ESC) [31] in which Wnt signaling is known to be activated and plays a key role in stem cell maintenance [57] or in placental development and differentiation [58]. Thus, epigenetic modifications leading to hypomethylation of the *HEMO* CpG‐rich promoter and activation of Wnt signaling could be considered as two concomitant events resulting in *HEMO* expression in tumors, stem cell, and placenta (Fig. 9).

In addition, we identified a specific pattern of *HEMO* expression in squamous tumors. Even though the Wnt pathway has been reported to play a role in squamous transdifferentiation [59], the specific underlying mechanisms leading to activation of *HEMO* in squamous (+/−keratinizing) tumor areas remained unclear and need to be further explored.

Lastly, *in silico* analysis provided evidence of a link between *HEMO* and immune signatures. Tumors with high *HEMO* transcript levels were found to be negatively associated with immune features such as regulation of T‐cell response or interferon response, particularly in lung, colon, and bladder tumors. This finding was unexpected as there is an accumulation of evidence showing endogenous retroviral products as triggers of antiviral immune response [60, 61, 62]. Nevertheless, *HEMO* belonging to an ERV with a degenerated 3′ LTR sequence, its contribution to dsRNA formation [63] that elicits in turn viral mimicry should be minimal. Furthermore, given that active Wnt/β‐catenin signaling has been documented as being a determinant factor of immune exclusion in tumors [64, 65, 66], frequent activation of this pathway in HEMO^High^ tumors may actually account for the observed downregulation of tumor immune response.

On the other hand, as a retroviral envelope protein, HEMO could still carry a functional immunosuppressive domain (ISD) that modulates the immune response [67]. Given that the shedding cleavage of the protein occurs inside the ISD sequence, regulation of the shedding process may have important consequences on the immunosuppressive function, both in physiological and tumor conditions.

## 5 Conclusion

Overall, our results strengthen the potential of HEMO as a tumor biomarker and therapeutic target. Because this retroviral envelope protein has the unique property of being shed and secreted into the blood, it is a promising serum marker for the detection and follow‐up of HEMO‐positive tumors, even in renal tumors where its expression in control adjacent tissues could be activated in the early stages of tumor development. In healthy individuals, the HEMO shed protein is filtered by the kidney, as we could not detect the protein in the urine, even in the 3rd trimester of pregnancy when its level in the serum is high ([31] and our unpublished results). Therefore, it could also be used as a urinary marker in case of renal injury or bladder carcinoma. In tumor sites other than kidney, depending on the relative levels of *HEMO* expression, tumors could be targeted by systemic anti‐HEMO therapy or solely by intra‐tumoral treatment to protect renal function. Given that this sequence codes for a membrane protein, HEMO is an interesting target for anti‐tumor strategies such as antibody‐drug‐conjugates (ADC) or oncolytic viruses in particular in tumors in which Wnt signaling is altered and where efficacy of immune checkpoint blockade is limited [68, 69].

## Conflict of interest

The authors declare no conflict of interest.

## Author contributions

AK was involved in all experimental strategy and design, generated and analyzed data and was involved in the writing/editing of the manuscript. AB designed and performed western blotting and qPCR, was involved in all data analyses and in writing/editing of the manuscript. OB participated in the design of the immunohistochemistry experiments and performed them. KDA initiated bioinformatics analysis on RNAseq data. BJ performed bioinformatics Gene Set Enrichment Analysis on RNAseq data. CM provided access to the MOSCATO cohort and participated in discussions. J‐YS participated in the design and analysis of immunohistochemistry experiments. TH supervised the study. OH conceptualized and directed the project, was involved in all strategy, design, analysis of data and writing/editing of the manuscript. All authors revised and approved the final version of the paper.

## Supporting information


**Fig. S1.** Expression level of *HERV‐env* genes in tumors of the TCGA cohorts. Boxplots of normalized (TPM) and log_2_‐transformed expression of *HERV‐env* genes in tumor (‘T’, orange) and control (‘C’, green) samples retrieved from TCGA‐Recount2. White boxes correspond to basal expression in normal (‘N’) tissues from GTEx‐Recount2. Data are shown as mean with 25–75th percentile range (box) and 10–90th percentile (whiskers). Mild outliers are depicted as black dots. P‐values are shown for the pairwise (N,T) comparison: *, p < 0.05; **, p < 0.01; ***, p < 0.001, Mann‐Whitney U‐test (see Table S3). (A‐D) Expression level of the 4 *HERV‐env* genes: *ERVW‐1* (ENSG00000242950), *ERVFRD‐1* (ENSG00000244476), *ERVV‐2* (ENSG00000268964) *env* and *ERV3‐1* (ENSG00000213462) genes. P‐values for pairwise comparisons between each group (T, C, N) are given in Table S3. HEMATO: Hematological tumors. (E) Expression level of *HEMO* and the 4 *HERV‐env* genes in the three kidney TCGA cohorts KICH, KIRC and KIRP.

**Fig. S2.** Association between *HEMO* expression and tumor stage, grade, histological type or molecular subtype. Boxplots of *HEMO* expression in TCGA tumors stratified by clinical/pathologic stages, neoplasm histologic grade, histological type and molecular subtype. Statistical significance was evaluated by the Mann‐Whitney U‐test for comparison of two groups, and by the Kruskal‐Wallis test for comparison of more than 2 groups (*, p < 0.05; **, p < 0.01; ***, p < 0.001). Significant p‐values are in red. Data are shown as mean with 25–75th percentile range (box) and 10–90th percentile (whiskers). Mild outliers are depicted as black dots. Cohorts are grouped according to body systems as in Fig. 1. ADC: Adenocarcinoma, ADSQ: Adenosquamous, BAC: Bronchoalveolar Carcinoma, NOS: Not Otherwise Specified, SC: Squamous Carcinoma, SCC: Squamous Cell Carcinoma, SPP: Solid Pattern Predominant.

**Fig. S3.** Comparison of *HEMO* expression within NCIH20, HCC827, OVMANA, Caco‐2, SW480 and HCT116 cell lines. Cell lines were selected among *HEMO* high expressing cases reported in [70]. Comparison was based on RT‐qPCR and western blot analysis (A), mutational status of *CTNNB1* and *APC* (B) and immunohistochemistry analysis (anti‐HEMO staining with 2F7 mAb) (C). Std: protein standard. Magnification: 40X, scale bar: 20 μm.

**Fig. S4.** *HEMO* upregulation is associated with active Wnt/β‐catenin pathway in COAD cohort. (A) GSEA enrichment plots showing ‘REACTOME formation of the beta catenin TCF transactivating complex’ (n°25 in Table S7) and ‘REACTOME TCF dependent signaling in response to Wnt’ (n°71 in Table S7) enriched signatures between COAD HEMO^High^ and HEMO^Low^ tumors. NES: Normalized Enrichment Score. (B) Heatmap for the significant Wnt‐related genes differentially expressed between HEMO^High^ and HEMO^Low^ tumors. Color gradation is representative of Log_2_ fold change. The differential level of *HEMO* expression is also indicated at the top of the heatmap (LogFC = 8). For all depicted genes (except for *APC* and *CTNNB1*) adjusted p‐value < 0.01. In bold, common upregulated genes found in UCEC cohort (Fig. 5D).

**Table S1.** Summary of immunohistochemistry protocols.

**Table S2.** List of the primers.

**Table S3.** List and size of the cohorts investigated in this study from GTEx and TCGA databases.

**Table S4.** Expression level of *HERV*‐*env* genes in male breast tumor samples.

**Table S5.** List of retroviral sequences annotated in the human genome (Ensembl).

**Table S6.** List of differentially‐expressed genes associated with *HEMO* in 8 TCGA cohorts (BLCA, BRCA, CESC, COAD, HNSC, LUAD, LUSC, UCEC).

**Table S7.** Results of Gene Set Enrichment Analysis (GSEA) performed on REACTOME and GO Biological process databases.

**Table S8.** Mutational status of *CTNNB1* and *APC* in HEMO^High^ and HEMO^Low^ tumors of representative TCGA cohorts.

**Table S9.** Top 10 GO:BP and Top 10 REACTOME gene sets for LUSC, COAD and BLCA cohorts.

## Data Availability

The original RNAseq data that support the findings in this study are openly available in Recount2 resource (https://jhubiostatistics.shinyapps.io/recount/), and the processed analyses are available in Tables [Link], [Link] in the supplementary material of this article.
