# Supplementary figures and images for: Therapeutic potential of the human endogenous retroviral envelope protein HEMO: a pan‐cancer analysis
Source: Mol Oncol. 2021 Oct 11;16(7):1451–73. doi: 10.1002/1878-0261.13069 (PMC8978518; doi:10.1002/1878-0261.13069)

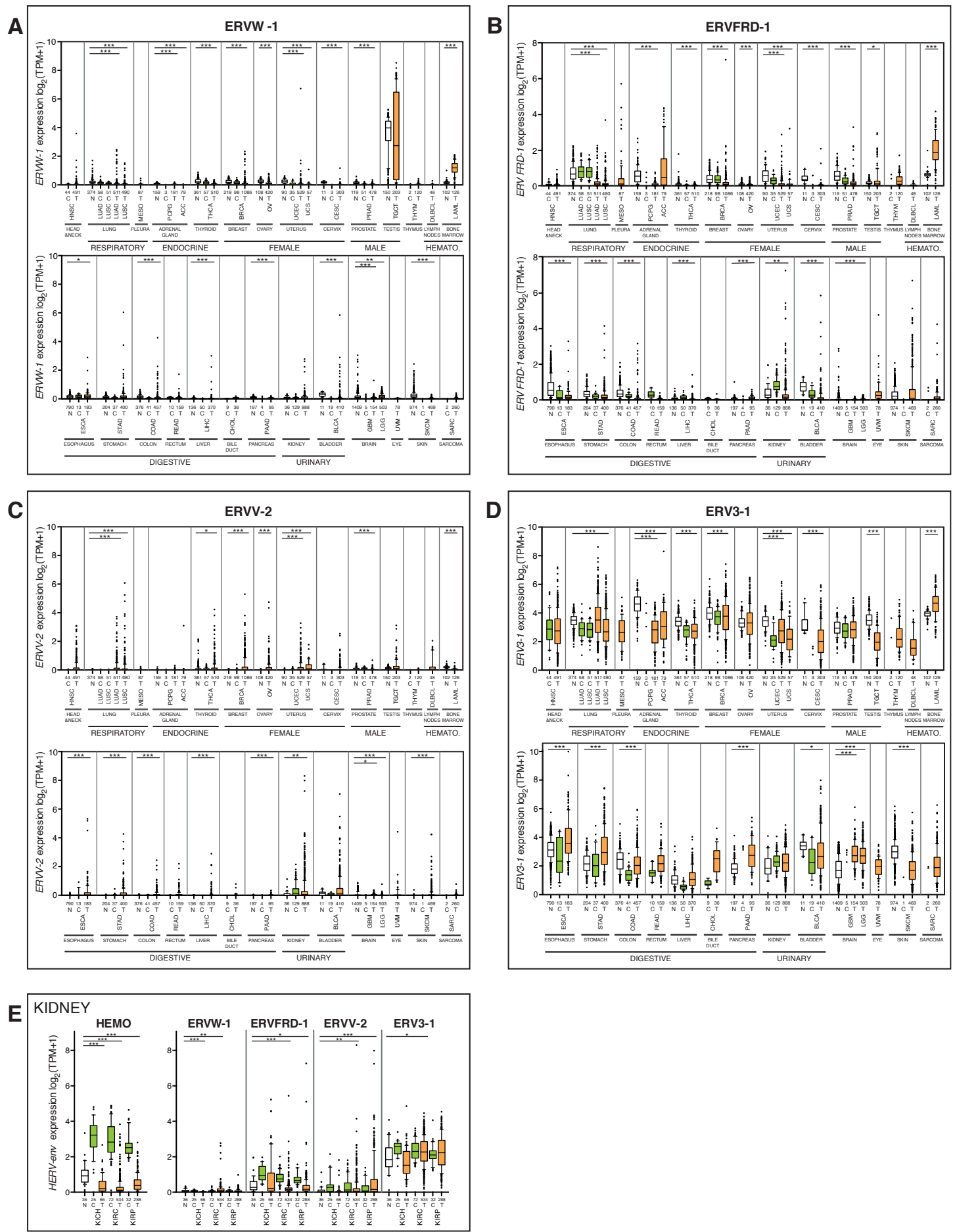

**Fig. S1.** Expression level of *HERV-env* genes in tumors of the TCGA cohorts.

Supplement: Supplementary file 1 — Fig. S1. Expression level of HERV‐env genes in tumors of the TCGA cohorts. Boxplots of normalized (TPM) and log2‐transformed expression of HERV‐env genes in tumor (‘T’, orange) and control (‘C’, green) samples retrieved from TCGA‐Recount2. White boxes correspond to basal expression in normal (‘N’) tissues from GTEx‐Recount2. Data are shown as mean with 25–75th percentile range (box) and 10–90th percentile (whiskers). Mild outliers are depicted as black dots. P‐values are shown for the pairwise (N,T) comparison: *, p < 0.05; **, p < 0.01; ***, p < 0.001, Mann‐Whitney U‐test (see Table S3). (A‐D) Expression level of the 4 HERV‐env genes: ERVW‐1 (ENSG00000242950), ERVFRD‐1 (ENSG00000244476), ERVV‐2 (ENSG00000268964) env and ERV3‐1 (ENSG00000213462) genes. P‐values for pairwise comparisons between each group (T, C, N) are given in Table S3. HEMATO: Hematological tumors. (E) Expression level of HEMO and the 4 HERV‐env genes in the three kidney TCGA cohorts KICH, KIRC and KIRP. [file MOL2-16-1451-s008.pdf]

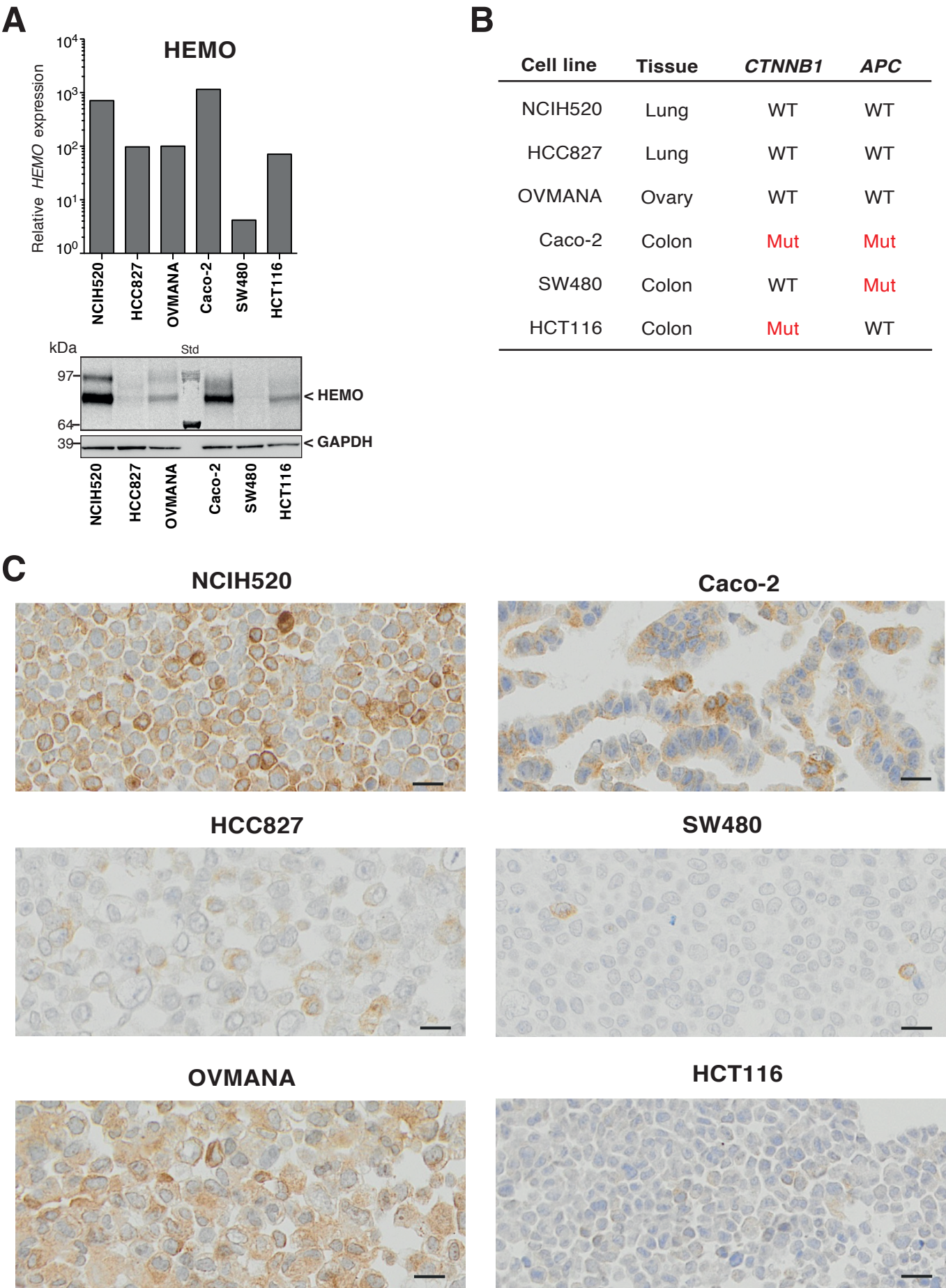

**Fig. S3.** Comparison of HEMO expression within NCIH20, HCC827, OVMANA, Caco-2, SW480 and HCT116 cell lines.

Supplement: Supplementary file 3 — Fig. S3. Comparison of HEMO expression within NCIH20, HCC827, OVMANA, Caco‐2, SW480 and HCT116 cell lines. Cell lines were selected among HEMO high expressing cases reported in [70]. Comparison was based on RT‐qPCR and western blot analysis (A), mutational status of CTNNB1 and APC (B) and immunohistochemistry analysis (anti‐HEMO staining with 2F7 mAb) (C). Std: protein standard. Magnification: 40X, scale bar: 20 μm. [file MOL2-16-1451-s011.pdf]

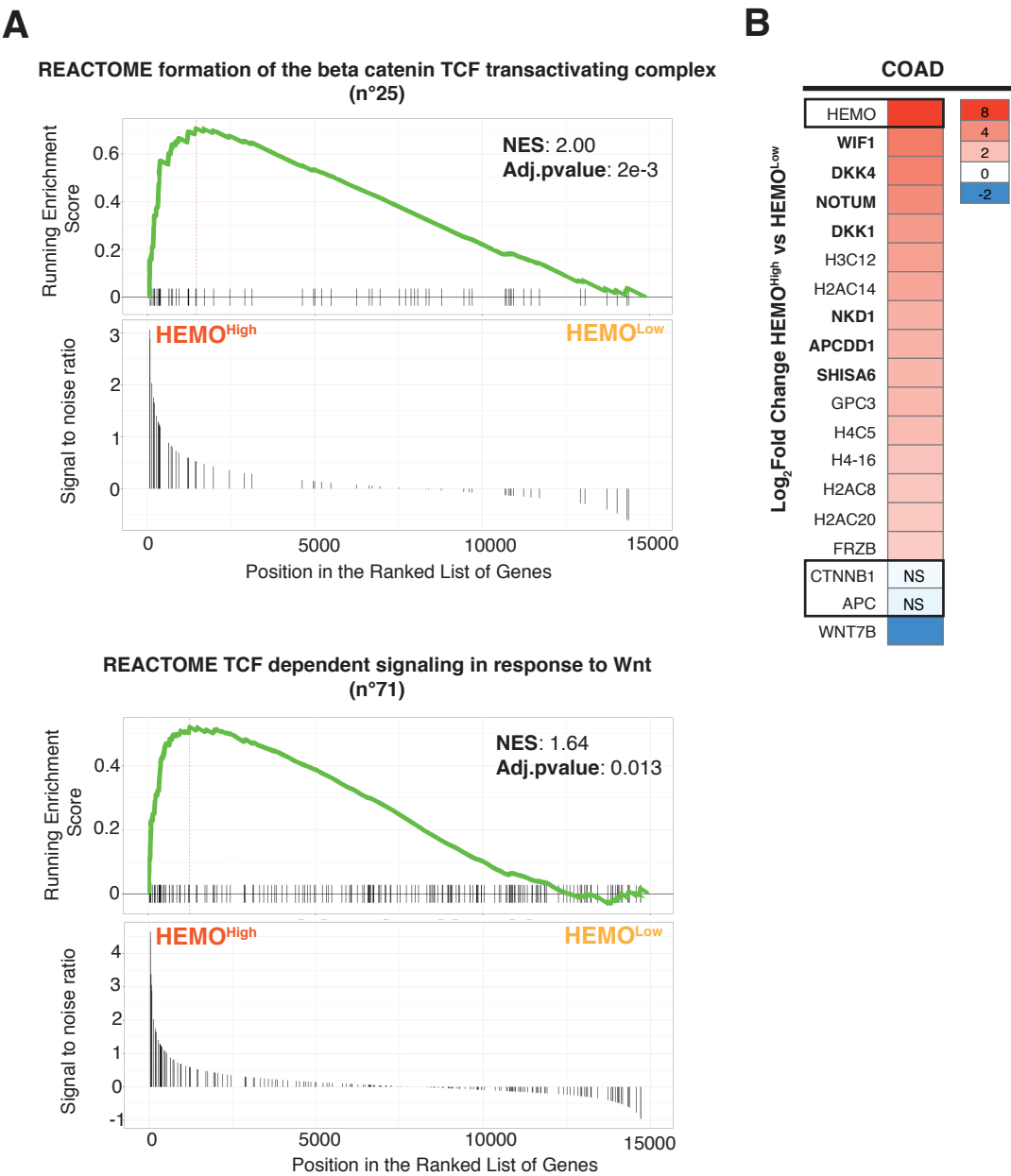

**Fig. S4.** *HEMO* upregulation is associated with active Wnt/ $\beta$ -catenin pathway in COAD cohort.

Supplement: Supplementary file 4 — Fig. S4. HEMO upregulation is associated with active Wnt/β‐catenin pathway in COAD cohort. (A) GSEA enrichment plots showing ‘REACTOME formation of the beta catenin TCF transactivating complex’ (n°25 in Table S7) and ‘REACTOME TCF dependent signaling in response to Wnt’ (n°71 in Table S7) enriched signatures between COAD HEMOHigh and HEMOLow tumors. NES: Normalized Enrichment Score. (B) Heatmap for the significant Wnt‐related genes differentially expressed between HEMOHigh and HEMOLow tumors. Color gradation is representative of Log2 fold change. The differential level of HEMO expression is also indicated at the top of the heatmap (LogFC = 8). For all depicted genes (except for APC and CTNNB1) adjusted p‐value < 0.01. In bold, common upregulated genes found in UCEC cohort (Fig. 5D). [file MOL2-16-1451-s013.pdf]
