# Supplementary material for: Therapeutic potential of the human endogenous retroviral envelope protein HEMO: a pan‐cancer analysis
Source: Mol Oncol. 2021 Oct 11;16(7):1451–73. doi: 10.1002/1878-0261.13069 (PMC8978518; doi:10.1002/1878-0261.13069)
Supplement: Supplementary file 2 — Fig. S2. Association between HEMO expression and tumor stage, grade, histological type or molecular subtype. Boxplots of HEMO expression in TCGA tumors stratified by clinical/pathologic stages, neoplasm histologic grade, histological type and molecular subtype. Statistical significance was evaluated by the Mann‐Whitney U‐test for comparison of two groups, and by the Kruskal‐Wallis test for comparison of more than 2 groups (*, p < 0.05; **, p < 0.01; ***, p < 0.001). Significant p‐values are in red. Data are shown as mean with 25–75th percentile range (box) and 10–90th percentile (whiskers). Mild outliers are depicted as black dots. Cohorts are grouped according to body systems as in Fig. 1. ADC: Adenocarcinoma, ADSQ: Adenosquamous, BAC: Bronchoalveolar Carcinoma, NOS: Not Otherwise Specified, SC: Squamous Carcinoma, SCC: Squamous Cell Carcinoma, SPP: Solid Pattern Predominant. [file MOL2-16-1451-s006.pdf]

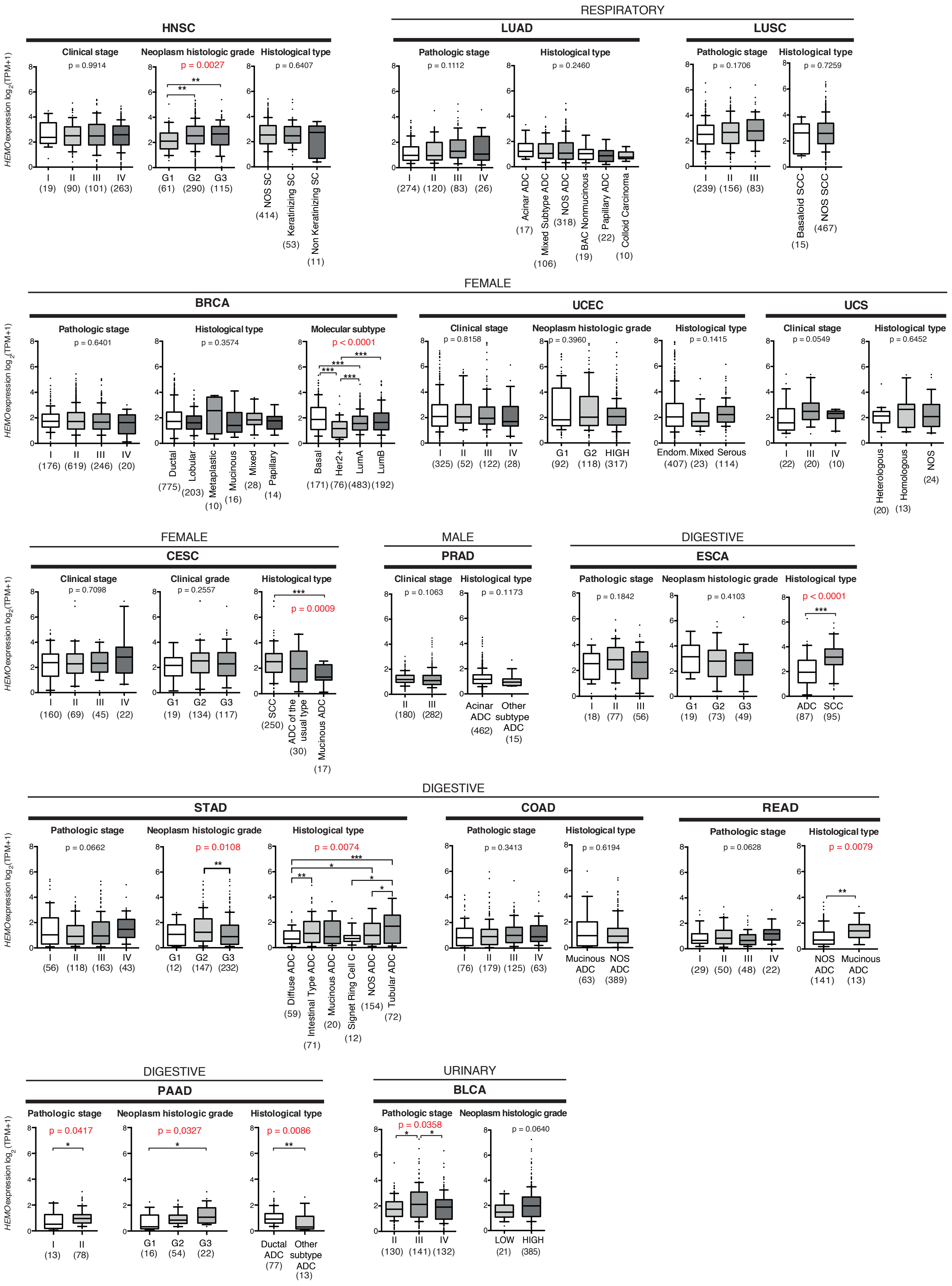

Fig. S2. Association between *HEMO* expression and tumor stage, grade, histological type or molecular subtype.
