# Supplementary material for: Therapeutic potential of the human endogenous retroviral envelope protein HEMO: a pan‐cancer analysis
Source: Mol Oncol. 2021 Oct 11;16(7):1451–73. doi: 10.1002/1878-0261.13069 (PMC8978518; doi:10.1002/1878-0261.13069)
Supplement: Supplementary file 5 — Table S1. Summary of immunohistochemistry protocols. [file MOL2-16-1451-s002.pdf]

Table S1: Summary of immunohistochemistry protocols

Protocol	Heat-induced antigen retrieval	Antibodies	Revelation
**HEMO**	ER2 buffer (pH9) 20 min,100°C	Mouse mAb 2F7 [31] 1 h at RT	Bond Polymer Refine Detection kit (Leïca Biosystems)
**HEMO/β-catenin**	ER2 buffer (pH9) 20 min,100°C	Mouse mAb 2F7 Mouse mAb anti-β-catenin (Dako, 1:800) 1 h each at RT	Bond Polymer Refine Red Detection (Leïca Biosystems) + HIGHDEF Green AP chromogen/substrate (Enzo Lifesciences) for β-catenin and Red chromogen (Leïca Biosystems) for HEMO
**HEMO/p63**	CC1 buffer (pH8) 76 min at 95°C	Mouse mAb anti-p63 (Dako, 1:20) 20 min, 36°C Mouse mAb anti-HEMO (2F7) 1 h RT	UltraView universal DAB detection kit (Roche) for HEMO and UltraView universal AP red (Roche) detection kit for p63
**CK5/6**	CC1 buffer (pH8) 36 min at 95°C	Mouse mAb anti-cytokeratins 5/6 (Dako, clone DC D5/16 B4, 1:40) 32 min at 36°C	UltraView universal DAB detection kit (Roche)
**CDX2**	CC2 buffer (pH6) 44 min at 95°C	Rabbit mAb anti-CDX2 (Zytomed, clone EPR2764Y, 1:200) 1 h at 36°C	UltraView universal DAB detection kit (Roche)
