# Supplementary material for: Therapeutic potential of the human endogenous retroviral envelope protein HEMO: a pan‐cancer analysis
Source: Mol Oncol. 2021 Oct 11;16(7):1451–73. doi: 10.1002/1878-0261.13069 (PMC8978518; doi:10.1002/1878-0261.13069)
Supplement: Supplementary file 6 — Table S2. List of the primers. [file MOL2-16-1451-s003.pdf]

Table S2: List of the primers

Primer name	Sequence
HEMO-F	5'-ACTATGGGCTCCCTTTCAAACT-3'
HEMO-R	5'-CATAGGAGGAAGTAGAGTGATT-3'
RPLP0-F	5'-GGCGACCTGGAAGTCCAACTA-3'
RPLP0-R	5'-CCATCAGCACCACAGCCTTC-3'
ASCT2-F	5'-GGCTTGGTAGTGTTTGCCAT-3'
ASCT2-R	5'-GGGCAAAGAGTAAACCCACA-3'
AXIN2-F	5'-GAGTGGACTTGTGCCGACTTCA-3'
AXIN2-R	5'-GGTGGCTGGTGCAAAGACATAG-3'
BAMBI-F	5'-AATGGATCGCCACTCCAGCTAC-3'
BAMBI-R	5'-ACACAGTGGGCAGCATCACAG-3'
CDX2-F	5'-ACAGTCGCTACATCACCATCCG-3'
CDX2-R	5'-CCTCTCCTTTGCTCTGCGGTTC-3'
CTNNB1-F	5'-TCTGAGGACAAGCCACAAGATTACA-3'
CTNNB1-R	5'-TGGGCACCAATATCAAGTCCAA-3'
DKK4-F	5'-CCAGAAAGTTCTGCCTCC-3'
DKK4-R	5'-CTTCTGCATGTGTGCCATCT-3'

Sequence of the primers used for RT-qPCR
