# Supplementary material for: Therapeutic potential of the human endogenous retroviral envelope protein HEMO: a pan‐cancer analysis
Source: Mol Oncol. 2021 Oct 11;16(7):1451–73. doi: 10.1002/1878-0261.13069 (PMC8978518; doi:10.1002/1878-0261.13069)
Supplement: Supplementary file 7 — Table S3. List and size of the cohorts investigated in this study from GTEx and TCGA databases. [file MOL2-16-1451-s012.pdf]

Table S3: List and size of the cohorts investigated in this study from GTEx and TCGA databases

SYSTEM	ORGAN		Acronym		Size N	Size C	Size T	Pairwise comparison (p-value) for HEMO: N vs T	C vs T	N vs C	Pairwise comparison (p-value) N vs T for other HERV-env: ERVW-1	ERVFRD-1	ERVV-2	ERV3-1
	**HEAD & NECK**	GTEx			na									
		TCGA	**HNSC**	Head and Neck Squamous Cell Carcinoma		44	491	na	< 0.0001	na	na	na	na	na
RESPIRATORY	**LUNG**	GTEx			374									
		TCGA	**LUAD**	Lung Adenocarcinoma		58	511	< 0.0001	0.0736	0.0322	< 0.0001	< 0.0001	< 0.0001	0.4803
			**LUSC**	Lung Squamous Cell Carcinoma		51	490	< 0.0001	< 0.0001	0.6701	< 0.0001	< 0.0001	< 0.0001	< 0.0001
	**PLEURA**	GTEx			na									
		TCGA	**MESO**	Mesothelioma		na	87	na	na	na	na	na	na	na
ENDOCRINE	**ADRENAL GLAND**	GTEx			159									
		TCGA	**PCPG**	Pheochromocytoma and Paraganglioma		3	181	< 0.0001	0.0008	0.3753	< 0.0001	< 0.0001	0.6742	< 0.0001
			**ACC**	Adrenocortical Carcinoma			79	0.0034	na	na	< 0.0001	0.7812	0.7382	< 0.0001
	**THYROID**	GTEx			361									
		TCGA	**THCA**	Thyroid Carcinoma		57	510	< 0.0001	< 0.0001	0.0002	< 0.0001	< 0.0001	0.0346	< 0.0001
FEMALE	**BREAST**	GTEx			218									
		TCGA	**BRCA**	Breast Invasive Carcinoma		98	1086	< 0.0001	0.0017	< 0.0001	< 0.0001	< 0.0001	< 0.0001	0.001
	**OVARY**	GTEx			108									
		TCGA	**OV**	Ovarian Serous Cystadenocarcinoma		na	420	0.0393	na	na	< 0.0001	0.0003	< 0.0001	0.3697
	**UTERUS**	GTEx			90									
		TCGA	**UCEC**	Uterine Corpus Endometrial Carcinoma		35	529	< 0.0001	< 0.0001	< 0.0001	< 0.0001	< 0.0001	< 0.0001	< 0.0001
			**UCS**	Uterine Carcinosarcoma			57	na	na	na	< 0.0001	< 0.0001	< 0.0001	< 0.0001
	**CERVIX**	GTEx			11									
		TCGA	**CESC**	Cervical Squamous Cell Carcinoma		3	303	< 0.0001	0.0010	0.4835	< 0.0001	< 0.0001	0.9601	0.0003
MALE	**PROSTATE**	GTEx			119									
		TCGA	**PRAD**	Prostate Adenocarcinoma		51	478	< 0.0001	0.0005	< 0.0001	< 0.0001	< 0.0001	< 0.0001	0.0553
	**TESTIS**	GTEx			150									
		TCGA	**TGCT**	Testicular Germ Cell Tumors		na	203	0.0804	na	na	0.8722	0.0159	0.6814	< 0.0001
	**THYMUS**	GTEx			na									
		TCGA	**THYM**	Thymoma		2	120	na	0.0740	na	na	na	na	na
HEMATOLOGICAL	**LYMPH NODES**	GTEx			na									
		TCGA	**DLBCL**	Diffuse Large B-cell Lymphoma		na	48	na	na	na	na	na	na	na
	**BONE MARROW**	GTEx			102									
		TCGA	**LAML**	Acute Myeloid Leukemia		na	126	< 0.0001	na	na	< 0.0001	< 0.0001	< 0.0001	< 0.0001
DIGESTIVE	**ESOPHAGUS**	GTEx			790									
		TCGA	**ESCA**	Esophageal Carcinoma		13	183	< 0.0001	< 0.0001	0.1542	0.0109	< 0.0001	< 0.0001	< 0.0001
	**STOMACH**	GTEx			204									
		TCGA	**STAD**	Stomach Adenocarcinoma		37	400	< 0.0001	< 0.0001	0.3745	0.1253	< 0.0001	< 0.0001	< 0.0001
	**COLON**	GTEx			376									
		TCGA	**COAD**	Colon Adenocarcinoma		41	457	< 0.0001	< 0.0001	< 0.0001	< 0.0001	< 0.0001	< 0.0001	< 0.0001
	**RECTUM**	GTEx			na									
		TCGA	**READ**	Rectum Adenocarcinoma		10	159	na	< 0.0001	na	na	na	na	na
	**LIVER**	GTEx			136									
		TCGA	**LIHC**	Liver Hepatocellular Carcinoma		50	370	< 0.0001	0.5852	0.0012	< 0.0001	< 0.0001	0.0003	0.5455
	**BILE DUCT**	GTEx			na									
		TCGA	**CHOL**	Cholangiocarcinoma		9	36	na	< 0.0001	na	na	na	na	na
	**PANCREAS**	GTEx			197									
		TCGA	**PAAD**	Pancreatic Adenocarcinoma		4	95	< 0.0001	0.7002	0.0444	< 0.0001	< 0.0001	< 0.0001	< 0.0001
URINARY	**KIDNEY**	GTEx			36									
		TCGA	**All**	Kidney Renal Cell Carcinoma		129	888	< 0.0001	< 0.0001	< 0.0001	0.2536	0.0019	0.0044	0.0591
			**KICH**	Kidney Chromophobe		25	66	< 0.0001			< 0.0001	0.9208	0.0538	0.0712
			**KIRC**	Kidney Renal Clear Cell Carcinoma		72	534	< 0.0001			0.001	0.0002	0.0084	0.0155
			**KIRP**	Kidney Renal Papillary Cell Carcinoma		32	288	< 0.0001			0.2552	0.0133	< 0.0001	0.0851
	**BLADDER**	GTEx			11									
		TCGA	**BLCA**	Bladder Urothelial Carcinoma		19	410	< 0.0001	< 0.0001	0.4346	< 0.0001	< 0.0001	0.139	0.0265
NERVOUS	**BRAIN**	GTEx			1409									
		TCGA	**GBM**	Glioblastoma Multiforme		5	154	< 0.0001	0.9278	0.0457	< 0.0001	0.6475	0.0217	< 0.0001
			**LGG**	Brain Lower Grade Glioma			503	< 0.0001	na	na	0.0049	< 0.0001	< 0.0001	< 0.0001
	**EYE**	GTEx			na									
		TCGA	**UVM**	Uveal Melanoma		na	78	na	na	na	na	na	na	na
	**SKIN**	GTEx			974									
		TCGA	**SKCM**	Skin Cutaneous Melanoma		1	469	< 0.0001	na	na	< 0.0001	0.3519	< 0.0001	< 0.0001
	**SARCOMA**	GTEx			na									
		TCGA	**SARC**	Sarcoma		2	260	na	0.7864	na	na	na	na	na

N: Normal tissue samples from GTEx. C: Control (Normal Adjacent Tumor) tissue samples and T: Tumor tissue samples from TCGA. na: not applicable. P-values correspond to pairwise comparison between tumor (T), control (C) and normal (N) groups for HEMO and between tumor (T) and normal (N) for the 4 HERV-env genes (Mann-Whitney U-test). Cohorts are grouped according to body systems as in Fig. 1. BRCA: 1074 female and 12 male tumors samples, SKCM: 103 primary and 366 metastatic unpaired samples.
