# Supplementary material for: Therapeutic potential of the human endogenous retroviral envelope protein HEMO: a pan‐cancer analysis
Source: Mol Oncol. 2021 Oct 11;16(7):1451–73. doi: 10.1002/1878-0261.13069 (PMC8978518; doi:10.1002/1878-0261.13069)
Supplement: Supplementary file 8 — Table S4. Expression level of HERV‐env genes in male breast tumor samples. [file MOL2-16-1451-s007.pdf]

Table S4: Expression level of *HERV-env* genes in male breast tumor samples

Male BRCA samples	Control	TUMOR				
	**HEMO**	**HEMO**	ERVW-1	ERVFRD-1	ERVV-2	ERV3-1
TCGA-AC-A62V		**0,094**	0,008	0,110	0,019	0,894
TCGA-AQ-A54O		**0,901**	0,014	0,033	0,000	1,854
TCGA-A8-A085		**0,952**	0,065	0,025	5,295	3,242
TCGA-BH-A0B4		**1,081**	0,000	0,021	0,000	3,136
TCGA-AO-A1KQ		**1,738**	0,031	0,089	1,014	2,456
TCGA-A1-A0SM		**1,788**	0,053	0,050	1,467	3,381
TCGA-AR-A1AV		**2,017**	0,214	0,049	0,044	5,540
TCGA-D8-A1XS		**2,172**	0,145	0,105	0,019	3,292
TCGA-EW-A1PD		**2,374**	0,115	0,123	0,063	4,111
TCGA-BH-A0DD	**0,127**	**2,617**	0,035	0,038	0,000	3,803
TCGA-E2-A14W		**3,711**	0,000	0,176	0,092	2,719
TCGA-EW-A6SA		**3,717**	0,034	0,012	0,000	2,842

Expression level (as log_2(TPM+1)) of *HEMO* and 4 other *HERV-env* genes, in 12 male tumor samples included in the BRCA cohort (1086 total samples)
