# Supplementary material for: Therapeutic potential of the human endogenous retroviral envelope protein HEMO: a pan‐cancer analysis
Source: Mol Oncol. 2021 Oct 11;16(7):1451–73. doi: 10.1002/1878-0261.13069 (PMC8978518; doi:10.1002/1878-0261.13069)
Supplement: Supplementary file 9 — Table S5. List of retroviral sequences annotated in the human genome (Ensembl). [file MOL2-16-1451-s010.pdf]

Table S5: List of retroviral sequences annotated in the human genome (Ensembl).

ENSEMBL "ERV" annotation	name	family	chromosome	aa	other name
**Envelope coding gene, full length (this study)**					
ENSG00000 226887	**ERVMER34-1**	endogenous retrovirus group MER34 member 1, envelope	Chromosome 4: 52,722,618-52,751,586	563 aa	HEMO
ENSG00000 242950	**ERVW-1**	endogenous retrovirus group W member 1, envelope	Chromosome 7: 92,468,380-92,477,986	538 aa	SYNCYTIN-1
ENSG00000 244476	**ERVFRD-1**	endogenous retrovirus group FRD member 1, envelope	Chromosome 6: 11,102,489-11,111,725	538 aa	SYNCYTIN-2
ENSG00000 268964	**ERVV-2**	endogenous retrovirus group V member 2, envelope	Chromosome 19: 53,044,740-53,051,680	535 aa	ENV-V2
ENSG00000 213462	**ERV3-1**	endogenous retrovirus group 3 member 1, envelope	Chromosome 7: 64,990,356-65,006,743	604 aa	ENV-R
**Others (truncated, lnc, unspecific)**					
ENSG00000 269526	**ERVV-1**	endogenous retrovirus group V member 1, envelope	Chromosome 19: 53,013,921-53,016,122	477 aa	ENV-V1
ENSG00000 267259	**ERVE-1**	endogenous retrovirus group E member 1	Chromosome 17: 28,232,590-28,235,281	lncRNA	
ENSG00000 251292	**ERVH-1**	endogenous retrovirus group H member 1	Chromosome 4: 23,723,262-23,733,579	lncRNA	
ENSG00000 233056	**ERVH48-1**	endogenous retrovirus group 48 member 1	Chromosome 21: 42,916,803-42,925,646	160 aa	SUPPRESSYN
ENSG00000 264801	**ERVFRD-3**	endogenous retrovirus group FRD member 3	Chromosome 9: 21,929,457-21,931,073	No protein	
ENSG00000 267696	**ERVK-28**	endogenous retrovirus group K member 28	Chromosome 19: 27,638,483-27,646,483	lncRNA	HERV-K(HML2)
ENSG00000 260565	**ERVK13-1**	endogenous retrovirus group K13 member 1	Chromosome 16: 2,660,348-2,682,379	lncRNA	HERV-K(HML2)
ENSG00000 142396	**ERVK3-1**	endogenous retrovirus group K3 member 1	Chromosome 19: 58,305,319-58,315,663	109 aa	HERV-K(HML6-1)
ENSG00000 269486	**ERVK9-11**	endogenous retrovirus group K9 member 11	Chromosome 19: 38,935,297-38,938,632	lncRNA	HERV-K(HML-3)
ENSG00000 230426	**ERVMER61-1**	long intergenic non-protein coding RNA 1036	Chromosome 1: 187,070,700-187,686,628	lncRNA	

Upper part: the five envelope retroviral sequences used in this study.
Bottom part: truncated or non-coding HERV sequences.
