# Supplementary material for: Therapeutic potential of the human endogenous retroviral envelope protein HEMO: a pan‐cancer analysis
Source: Mol Oncol. 2021 Oct 11;16(7):1451–73. doi: 10.1002/1878-0261.13069 (PMC8978518; doi:10.1002/1878-0261.13069)
Supplement: Supplementary file 12 — Table S8. Mutational status of CTNNB1 and APC in HEMOHigh and HEMOLow tumors of representative TCGA cohorts. [file MOL2-16-1451-s004.pdf]

Table S8: Mutational status of *CTNNB1* and *APC* in HEMO^{High} and HEMO^{Low} tumors of representative TCGA cohorts.

		HEMO **subgroup**		***CTNNB1***		***APC***	
	Cohort	Size	Expression level (TPM)	mut (%)	p-value	mut (%)	p-value
	**HNSC**	High (49)	> 12.2	2	1.000	4	1.000
		Low (49)	< 1.1	0		2	
		All (491)		0.2		0.8	
RESPIRATORY	**LUAD**	High (51)	> 5.6	3.9	0.4950	5.9	0.6175
		Low (51)	< 0.3	0		2	
		All (511)		2.3		1.8	
	**LUSC**	High (49)	> 14.0	0	1.000	2	1.000
		Low (49)	< 1.0	0		0	
		All (490)		0		1.6	
FEMALE	**BRCA**	High (111)	> 6.7	0	1.000	0	1.000
		Low (111)	< 0.5	0		0	
		All (1086)		0		0.3	
	**UCEC**	High (53)	> 18.6	73.6	< 0.0001	5.7	0.7157
		Low (53)	< 0.7	1.9		9.4	
		All (529)		18.3		7.2	
	**CESC**	High (30)	> 11.5	10	0.2373	0	1.000
		Low (30)	< 0.6	0		3.3	
		All (303)		1.3		0.7	
DIGESTIVE	**COAD**	High (46)	> 3.1	4.3	1.000	67.4	0.0016
		Low (46)	< 0.06	6.5		32.6	
		All (457)		3.7		60.6	
URINARY	**BLCA**	High (41)	> 8.4	12.2	0.0549	0	1.000
		Low (41)	< 0.4	0		2.5	
		All (410)		2		2.4	

Numbers of samples in each subgroup, corresponding to 90th (HEMO^{High}) and 10th (HEMO^{Low}) percentiles of the TCGA cohorts, are indicated in brackets with corresponding minimal and maximal *HEMO* expression level (TPM). Fischer's exact test. Gray line: size of the cohorts (All) is indicated, together with the global % of mutations of the two genes.
