# Supplementary material for: Therapeutic potential of the human endogenous retroviral envelope protein HEMO: a pan‐cancer analysis
Source: Mol Oncol. 2021 Oct 11;16(7):1451–73. doi: 10.1002/1878-0261.13069 (PMC8978518; doi:10.1002/1878-0261.13069)
Supplement: Supplementary file 13 — Table S9. Top 10 GO:BP and Top 10 REACTOME gene sets for LUSC, COAD and BLCA cohorts. [file MOL2-16-1451-s001.pdf]

Table S9: Top 10 GO:BP and Top 10 REACTOME gene sets for LUSC, COAD and BLCA cohorts

LUSC	GO:BP	LUSC	REACTOME
1	GO regulation of cell activation	1	**REACTOME neutrophil degranulation**
2	GO metal ion homeostasis	2	**REACTOME interferon signaling**
3	**GO response to bacterium**	3	**REACTOME immunoregulatory interactions between a Lymphoid and a non-Lymphoid cell**
4	**GO leukocyte differentiation**	4	**REACTOME interferon gamma signaling**
5	GO regulation of GTPase activty	5	**REACTOME costimulation by the CD28 family**
6	GO regulation of hemopoiesis	6	**REACTOME chemokine receptors bind chemokines**
7	GO positive regulation of response to external stimulus	7	**REACTOME complement cascade**
8	**GO T cell activation**	8	**REACTOME interleukin-2 family signaling**
9	**GO activation of immune response**	9	REACTOME generation of second messenger molecules
10	**GO regulation of lymphocyte activation**	10	**REACTOME PD1 signaling**
**COAD**	**GO:BP**	**COAD**	**REACTOME**
1	GO mRNA processing	1	**REACTOME PD-1 signaling**
2	GO ribonucleoprotein complex biogenesis	2	REACTOME generation of second messenger molecules
3	GO RNA splicing	3	**REACTOME interferon alpha/beta signaling**
4	GO RNA splicing via transesterification reactions	4	**REACTOME costimulation by the CD28 family**
5	GO ribonucleoprotein complex subunit organization	5	**REACTOME interferon gamma signaling**
6	GO spliceosomal complex assembly	6	REACTOME DNA replication pre-initiation
7	GO spliceosomal snRNP assembly	7	**REACTOME immunoregulatory interactions between a Lymphoid and a non-Lymphoid cell**
8	GO mRNA cis splicing via spliceosome	8	REACTOME RNA polymerase II transcribes snRNA genes
9	GO mRNA splice site selecion	9	**REACTOME antigen processing cross presentation**
10	GO spliceosomal tri-snRNP complex assembly	10	**REACTOME MHC class II antigen presentation**
**BLCA**	**GO:BP**	**BLCA**	**REACTOME**
1	**GO myeloid leukocyte mediated immunity**	1	REACTOME hemostasis
2	GO cellular ion homeostasis	2	REACTOME signaling by GPCR
3	GO regulation of cell activation	3	**REACTOME neutrophil degranulation**
4	GO metal ion homeostasis	4	REACTOME GPCR ligand binding
5	GO regulation of ion transport	5	**REACTOME Leishmania infection**
6	**GO response to bacterium**	6	REACTOME muscle contraction
7	**GO leukocyte differentiation**	7	**REACTOME TCR signaling**
8	GO regulation of hemopoiesis	8	REACTOME SRP dependent cotranslational protein targeting to membrane
9	GO circulatory system process	9	**REACTOME anti-inflammatory response favouring Leishmania parasite infection**
10	GO regulation of system process	10	**REACTOME immunoregulatory interactions between a Lymphoid and a non-Lymphoid cell**

Data are extracted from Table S7. Immune-related gene sets are depicted in blue.
